# Turning publicly available gene expression data into discoveries using gene set context analysis

**DOI:** 10.1093/nar/gkv873

**Published:** 2015-09-08

**Authors:** Zhicheng Ji, Steven A. Vokes, Chi V. Dang, Hongkai Ji

**Affiliations:** 1 Department of Biostatistics, Johns Hopkins University Bloomberg School of Public Health, 615 North Wolfe Street, Baltimore, MD 21205, USA; 2 Department of Molecular Biosciences, The University of Texas at Austin, 2500 Speedway Stop A4800, Austin, TX 78712, USA; 3 Institute for Cellular and Molecular Biology, The University of Texas at Austin, 2500 Speedway Stop A4800, Austin, TX 78712, USA; 4 Abramson Cancer Center, University of Pennsylvania, 3400 Spruce Street, Philadelphia, PA 19104, USA

## Abstract

Gene Set Context Analysis (GSCA) is an open source software package to help researchers use massive amounts of publicly available gene expression data (PED) to make discoveries. Users can interactively visualize and explore gene and gene set activities in 25,000+ consistently normalized human and mouse gene expression samples representing diverse biological contexts (e.g. different cells, tissues and disease types, etc.). By providing one or multiple genes or gene sets as input and specifying a gene set activity pattern of interest, users can query the expression compendium to systematically identify biological contexts associated with the specified gene set activity pattern. In this way, researchers with new gene sets from their own experiments may discover previously unknown contexts of gene set functions and hence increase the value of their experiments. GSCA has a graphical user interface (GUI). The GUI makes the analysis convenient and customizable. Analysis results can be conveniently exported as publication quality figures and tables. GSCA is available at https://github.com/zji90/GSCA. This software significantly lowers the bar for biomedical investigators to use PED in their daily research for generating and screening hypotheses, which was previously difficult because of the complexity, heterogeneity and size of the data.

## INTRODUCTION

**Figure 1.**
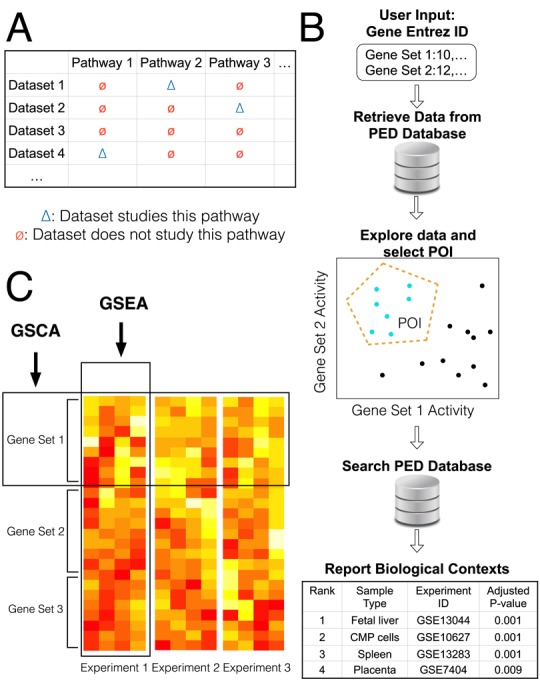
Gene Set Context Analysis. (**A**) Data generated by one investigator for studying one pathway (blue triangle) may also contain information about other pathways (red circles). This information has not been fully utilized so far. (**B**) GSCA takes one or more gene sets as input. Users specify a combinatorial expression pattern of interest (POI) of these gene sets. GSCA then searches a large compendium of publicly available gene expression data and identifies all enriched biological contexts associated with the POI. (**C**) Comparison between GSCA and GSEA. GSEA analyzes thousands of gene sets sequentially in one data set, while GSCA analyzes one or multiple gene sets across massive amounts of samples from many data sets.

Publicly available gene expression data (PED) are an invaluable resource for biomedical research. There are currently over 1,000,000 microarray and high-throughput sequencing samples stored in public databases such as the Gene Expression Omnibus (GEO) (1) and ArrayExpress (2). These include at least 200,000+ gene expression samples. These databases, which are continuing to quickly expand, contain vast amounts of information that have yet to be fully utilized. For instance, microarray data generated by one investigator for studying pathway A may also contain information about pathway B. This information may not be used by the original investigator for his/her study of pathway A, but it can be useful for other people who want to study pathway B (Figure 1A).

A unique feature of PED is that it contains samples contributed by scientists worldwide, covering a wide variety of biological contexts including different cells, tissues and disease types, different developmental time points and different stimuli, etc. Thus, if there is a convenient way to reuse the data, one will be able to systematically examine gene or pathway's activities in a broad spectrum of biological contexts, which would not be possible if an investigator had to rely on him- or herself to generate all the data. However, several obstacles impede the usage of PED for data mining, including data normalization, annotation, visualization and retrieval. In addition, it is technically challenging to meaningfully analyze the data and turn them into useful knowledge. Unfortunately, none of these are trivial given the complexity, heterogeneity and size of the data. To help researchers effectively use PED in their daily research, we developed Gene Set Context Analysis (GSCA) to allow them to conveniently explore gene and gene set activities in a large collection of normalized and annotated GEO microarray samples and to systematically link gene set activities to biological contexts.

GSCA is constructed based on 25,000+ human and mouse samples representing 1000+ different biological contexts. By providing one or multiple genes or gene sets as input, users can interactively examine their transcriptional activities in these samples. Users can also specify a gene set activity pattern of interest (POI) and query the expression compendium to systematically identify biological contexts associated with the specified pattern (Figure 1B). This analysis allows one to answer questions such as ‘which diseases are associated with high activity of pathway A, low activity of pathway B and medium activity of pathway C’. It can help researchers with new gene sets (e.g. gene sets obtained from a high-throughput experiment) to quickly extend their discoveries via finding previously unknown biological contexts of gene set functions. GSCA has a graphical user interface (GUI). Using the GUI, users can conveniently visualize the data, customize the analyses, and save analysis results and plots for publications.

GSCA is conjugated to Gene Set Enrichment Analysis (GSEA, Figure 1C) (3). GSEA is designed to analyze association between gene sets and biological signals in one data set. For example, given a microarray data set, GSEA can analyze thousands of gene sets one-by-one to identify which gene sets are enriched in differentially expressed genes in that data set. Unlike GSEA, GSCA analyzes expression levels of one or multiple gene sets in massive amounts of samples from thousands of data sets. The analysis aims to systematically identify biological contexts in which the input gene sets show user-specified expression patterns.

GSCA can be viewed as a generalization of ChIP-PED, a method we devised to support the analysis of genome-wide chromatin immunoprecipitation (i.e. ChIP-seq and ChIP-chip) data (4). ChIP-PED analyzes the expression of a transcription factor (TF) and its target gene set in PED in order to find the biological contexts associated with the TF function. It was originally motivated by our needs for characterizing *cMyc* function (5). A limitation of ChIP-PED is that it can only analyze a TF and a target gene set of the TF. Thus, it represents a special case of GSCA with only two input gene sets. One cannot use it to analyze other number and other types of gene sets. Moreover, ChIP-PED only considers high or low expression and cannot analyze more complex expression patterns. ChIP-PED does not have a GUI. All analyses have to be performed by typing commands via a keyboard which makes data exploration inconvenient. This has greatly limited the use of ChIP-PED by biologists. These limitations motivated the development of GSCA which generalizes ChIP-PED by allowing one to analyze any kind and any number of gene sets through a user-friendly interface. The GUI allows one to interactively specify and analyze highly sophisticated expression patterns. It also allows users to conveniently import gene sets and PED compendia, visualize data, tune parameters, perform cross-species analyses, and export analysis results and plots. As we shall demonstrate below, these new functions are crucial for effectively using the highly complex public expression data.

Previously, a number of other methods and tools have also been developed for using PED in different ways. For example, PED has been used to build the gene expression BARCODE for predicting tissue type of microarray samples (6), tools for predicting genes related to specific biological processes (7,8), phenome-genome network (9), functional map of human genome (10), and tools for phenotypic profiling (11) and disease diagnosis (12). It has also been used to study the global gene expression characteristics in human (13) and improve transcription factor target gene prediction (14). Most of these tools, however, are developed for purposes different from GSCA and do not support query and analyses of expression levels of gene sets and their association with biological contexts. The Expression Atlas (15) is a tool that allows users to query differentially expressed genes in a large number of curated PED data sets. It can also be used to query gene's baseline expression in healthy or untreated conditions. However, unlike GSCA, one cannot search the Expression Atlas using user-specified gene set activity patterns (e.g. an arbitrary combinatorial expression pattern of multiple genes or gene sets) and identify their associated biological contexts.

## MATERIALS AND METHODS

### Gene set context analysis

The primary aim of GSCA is to link gene set activities to biological contexts. Hereinafter, gene set is a general concept. A single gene is viewed as a gene set with only one gene.

Currently, GSCA is constructed based on four compendia of annotated PED samples compiled by the Gene Expression BARCODE project (16): a compendium of 11,778 samples from Affymetrix Human Genome U133A Array (GPL96), a compendium of 5,153 samples from Affymetrix Human Genome U133 Plus 2.0 Array (GPL570), a compendium of 313 samples from Affymetrix Human Genome U133A 2.0 Array (GPL571) and a compendium of 9,444 samples from Affymetrix Mouse Genome 430 2.0 Array (GPL1261). Samples within each compendium are from the same microarray platform. For each array platform, BARCODE collected its samples from GEO and consistently normalized them using frozen RMA (fRMA) (17). Gene expression levels were then characterized using BARCODE *z*-scores which were determined by modeling each gene's expression values across all samples to adjust for probe effects (16). Z-scores close to zero represent absent or low expression, whereas large z-scores correspond to high expression. Previous studies have shown that after data are processed using this protocol, the unwanted variation such as lab and batch effects within each PED compendium usually is smaller than the biological variation across diverse sample types, making it feasible to meaningfully compare genes’ expression levels across heterogeneous samples (4,16). Note, however, that expression values from different platforms are not directly comparable. The biological contexts, defined as samples’ cell or tissue types and associated treatment or disease conditions, were derived from BARCODE annotations as previously described (4,16) (Supplementary Materials). Each sample is associated with one context. Not all samples have been annotated in BARCODE. The four compendia above represent the annotated subset of BARCODE 3.0 samples. These samples and annotations provide a good coverage of major tissues and cell types in human and mouse (Supplementary Materials, Supplementary Table S1).

Consider *S* gene sets and a compendium of *N* normalized gene expression samples. Suppose the samples are annotated with a total of *C* biological contexts. Let *x*_*gi*_ denote the expression level of gene *g* in sample *i* (*i* ∈ 1, ..., *N*). We define the activity of gene set *s* (*s* ∈ 1, ..., *S*) in sample *i*, *y*_*si*_, as a weighted average of expression levels of individual genes in the gene set:

(1) \begin{equation*} y_{si} = \frac{ \sum _{g \in s} {w_{g} x_{gi}} }{\sum _{g \in s} |w_{g}| } \end{equation*}

where *ω*_*g*_s are user-specified weights. If *ω*_*g*_ = 1 for all *g* (default setting), the gene set activity is the mean expression level of all genes in the gene set. Negative weights can be used to handle genes whose expressions are expected to be anti-correlated. For instance, for a gene set consisting of target genes of a TF, one can assign *ω*_*g*_ = 1 to genes activated by the TF and *ω*_*g*_ = −1 to genes repressed by the TF. *y*_*si*_ defined in this way does not measure the mean expression level of target genes. However, it provides a measure that can be used to compare the regulatory activities of the TF across samples. As an example, consider a TF target gene set *s* consisting of one positive (activated) target and one negative (repressed) target. Suppose in sample *i*, the TF is highly active, its positive target (*ω*_*g*_ = 1) has high expression 10, and its negative target (*ω*_*g*_ = −1) has low expression −2. Correspondingly, the target gene set activity *y*_*si*_ = [1 × 10 + (−1) × (− 2)] / [|1| + |−1|] = 6. Suppose in another sample *k*, the TF is expressed at low levels, its positive target is inactive and has low expression 1, and its negative target is not repressed and has high expression 9. As a result, the target gene set activity *y*_*sk*_ = [1 × 1 + (−1) × 9] / [|1| + |−1|] = −4. Here, *y*_*si*_ > *y*_*sk*_ is consistent with the fact that the TF is more actively executing its regulatory role (i.e. activating its positive target and repressing its negative target) in sample *i* compared to sample *k*. This shows that the gene set activity defined in this way can be used to compare the regulatory activities of a TF across samples. By contrast, if one sets *ω*_*g*_ = 1 for both target genes, then *y*_*si*_ becomes the mean expression of target genes, which cannot reflect the TF's regulatory activity since *y*_*si*_ = (10 − 2) / 2 = 4, *y*_*sk*_ = (1 + 9) / 2 = 5, and *y*_*si*_ < *y*_*sk*_. A more concrete example to illustrate this is provided in Supplementary Materials and Supplementary Figure S1.

After gene set activities are defined, one can specify a gene set activity POI. For instance, if one is interested in samples with ‘high gene set 1 activity and low gene set 2 activity’, the POI may be defined as ‘*y*_1*i*_ > *c*_1_ and *y*_2*i*_ < *c*_2_’ where *c*_1_ and *c*_2_ are two user-chosen cutoffs. Complex POIs can be specified interactively using the GUI, or using formulas that describe relationships among different gene sets (see examples below).

Once the POI is specified, GSCA will search the PED compendium to find samples with the POI. It then evaluates which biological contexts are significantly associated with the POI. Each biological context can have multiple samples. Suppose context *c* has *n*_*c*_ samples, among which *k*_*c*_ have the POI. GSCA compares *n*_*c*_ and *k*_*c*_ with the total number of compendium samples *N* and the total number of samples with the POI *K* using a Fisher's exact test to determine whether context *c* is enriched in samples with the POI. *P*-values from the *C* hypothesis tests are Bonferroni corrected to adjust for multiple testing. For each context, a POI fold change is computed as:

(2) \begin{equation*} f_c = \frac{(k_c+K/N)/(n_c+1)}{K/N} \end{equation*}

Biological contexts with adjusted *P*-value and fold change passing a user-specified cutoff (adjusted *P*-value <0.05 and fold change >1.5 by default) are reported.

Conceptually, GSCA is analogous to BLAST (18). BLAST searches a sequence database based on sequence similarity, whereas GSCA searches a gene expression database to look for user-specified gene set activities. This search can often lead to new discoveries. For instance, suppose one obtained a new gene set from a costly experiment, one can use GSCA to quickly discover previously unknown relationship between the new gene set and diseases not surveyed by the original experiment.

### Software

**Figure 2.**
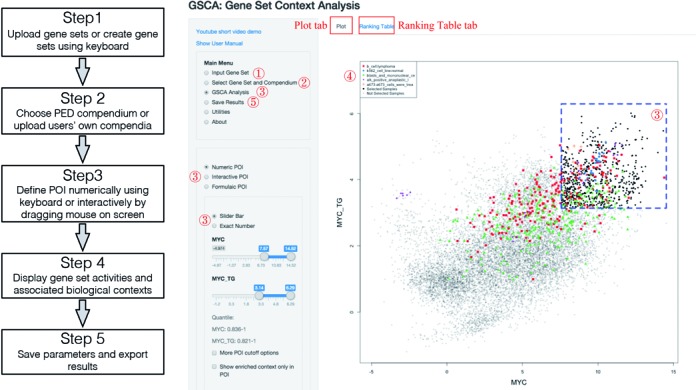
GSCA analysis pipeline. A default GSCA work flow is shown on the left. A screenshot of GSCA interface is shown on the right. Functions in the user interface corresponding to the five analysis steps are indicated using the red numbers.

GSCA is developed using the statistical programming language R. The shiny package in R is used to develop the GUI. The software and GUI provide a variety of functions to support the five major steps of data analysis shown in Figure 2. These include functions for uploading gene sets or creating them using a keyboard, choosing PED compendia or uploading users’ own compendia, defining POI (either numerically by entering values or formulas using a keyboard, or interactively by dragging a mouse on the computer screen), displaying gene set activities using histograms, scatter plots or heat maps, saving analysis parameters and results, exporting plots into publication quality images, and numerous utility functions such as converting gene sets between species to support cross-species analyses. These functions will be explained and demonstrated in detail below through examples. GSCA can be run on Windows, Linux and Mac operating systems. Information on how to install GSCA is provided in Supplementary Materials.

## RESULTS

### Example I: demonstration of GSCA using two gene sets

**Figure 3.**
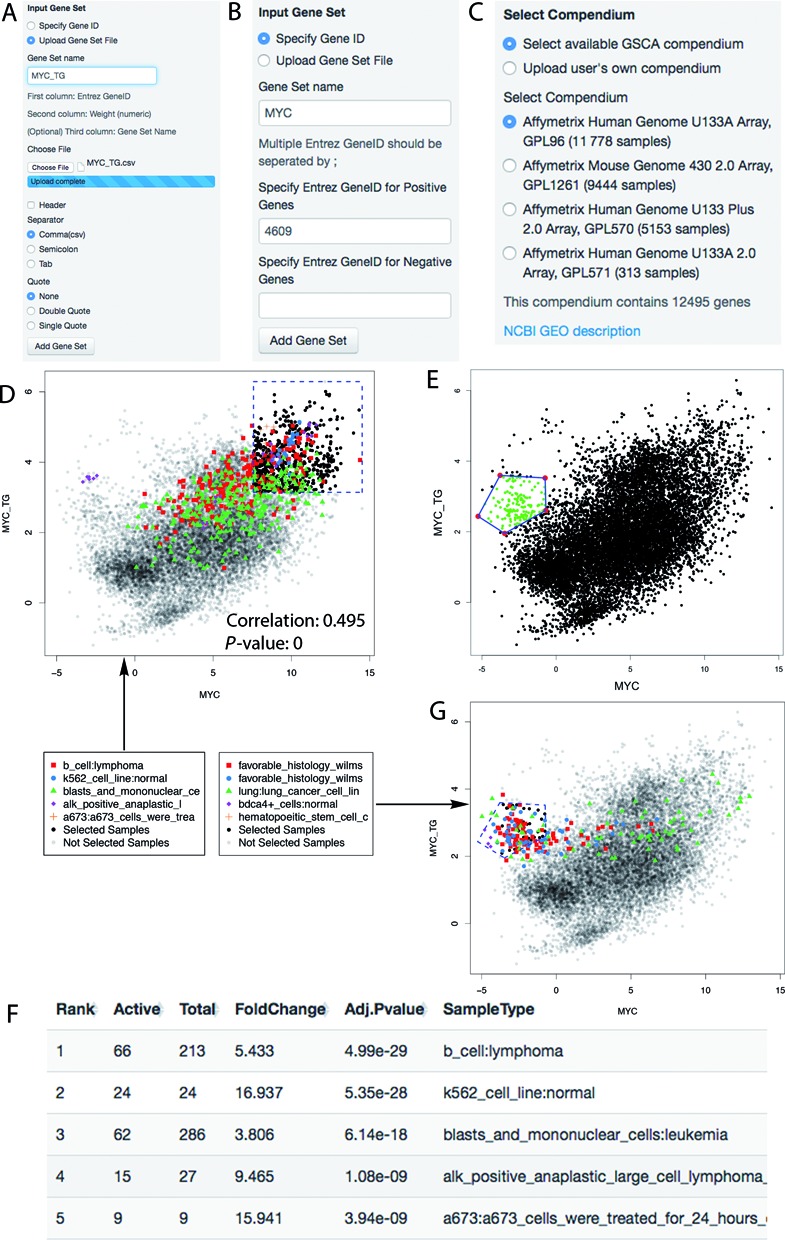
GSCA demonstrated using the MYC example. (**A**) Panel for uploading gene set files. (**B**) Panel for inputting ENTREZ gene IDs. (**C**) Panel for selecting available compendia or uploading users’ own compendium. (**D**) GSCA scatter plot showing the default numeric POI. (**E**) POI defined interactively by drawing a polygon. (**F**) Ranking table reported by GSCA showing the top biological contexts associated with the numeric POI. (**G**) Scatter plot that highlights samples from five most significant biological contexts in the analysis of POI interactively specified in (**E**).

We first demonstrate the software in detail using an analysis of MYC and its target genes (Figure 3). MYC is a transcription factor involved in ∼30% of human tumors. Previously, Ji *et al*. identified a core set of 51 target genes of human MYC by analyzing gene expression, ChIP-chip and ChIP-seq data from embryonic stem cells and several cancer cell lines (5). Ji *et al*. speculated that these genes may provide a signature for MYC TF activity, and they used this signature to search for diseases where MYC may play a functional role. Here we illustrate how this analysis can be done using the GSCA software.

#### Step 1: input gene set

First, choose ‘Input Gene Set’ in the main menu (Figure 2). The input for this analysis consists of two gene sets: one is MYC and the other one is its target genes. According to Ji *et al*., the 51 target genes are all activated by MYC, therefore all weights are set to *ω*_*g*_ = 1. We saved the input gene sets (specified using ENTREZ Gene Identifiers (IDs)) and weights into a single text file (Supplementary Table S2). This file can be directly loaded into GSCA via ‘Upload Gene Set File’ function (Figure 3A). The input file can contain multiple gene sets, and the weights can be any positive or negative numbers and are not required to be integers. Users can analyze their own gene sets by preparing input files following the same format of Supplementary Table S2.

An alternative way to input gene sets is to use a keyboard. To do so, one first selects the ‘Specify Gene ID’ function. One can then type ENTREZ Gene IDs and create a name for each gene set (Figure 3B). ENTREZ IDs entered in the ‘Positive genes’ box receive weight *ω*_*g*_ = 1, and those in the ‘Negative genes’ box receive weight *ω*_*g*_ = −1. This approach does not allow one to use other weight values.

#### Step 2: select gene set and a PED compendium

By choosing ‘Select Gene Set and Compendium’ in the main menu (Figure 2), one will move to the next step. In this step, one can select some or all input gene sets for analysis. One also needs to specify a PED compendium (Figure 3C). Users can either select a compendium available in GSCA, or upload their own PED compendium (instructions on how to prepare users’ own compendium are shown on the GSCA GUI). For demonstration purpose, we choose the compendium of Affymetrix Human Genome U133A Array (GPL96) for the MYC analysis.

#### Step 3: explore data and define gene set activity pattern of interest

Next, one can select ‘GSCA Analysis’ in the main menu (Figure 2). Depending on the number of analyzed gene sets, the software will display gene set activities across all compendium samples on the screen using histograms (for 1 gene set), scatter plots (for 2 gene sets), or heat maps (for >2 gene sets). The MYC analysis involves two gene sets. Therefore, a scatter plot is generated to show MYC expression (i.e. its RNA level) and its target gene activity in compendium samples (Figure 3D). The plot shows a clear positive correlation between MYC and its target gene activity (Pearson correlation = 0.495, *P*-value = 0 based on correlation test (19)). The degree of correlation is non-trivial considering the heterogeneous lab and cell type origins of these samples. This demonstrates the data quality and the feasibility to meaningfully compare gene set activities across heterogeneous PED samples.

In the same ‘GSCA Analysis’ menu, one can now specify a POI. POI can be defined numerically, interactively or using formulas.

If one chooses ‘Numeric POI’ (Figure 2), the POI will be defined in the form of ‘gene set 1 ∈ (*c*_11_, *c*_12_), and gene set 2 ∈ (*c*_21_, *c*_22_),..., and gene set S ∈ (*c*_*S*1_, *c*_*S*2_)’. The cutoffs *c*_*sl*_ can be chosen to correspond to certain standard deviations (SDs) away from the mean of *y*_*si*_ across all PED samples, or certain quantiles, or certain *P*-values based on a normal distribution fitted to the data. When *c*_*s*1_ is set to be the smallest possible value in the PED compendium, *y*_*si*_ ∈ (*c*_*s*1_, *c*_*s*2_) reduces to *y*_*si*_ < *c*_*s*2_. Similarly, *y*_*si*_ > *c*_*s*1_ can be represented by setting *c*_*s*2_ to the largest possible value in the compendium. By default, GSCA defines POI as ‘*y*_1*i*_ > *c*_1_, *y*_2*i*_ > *c*_2_, ..., *y*_*Si*_ > *c*_*S*_’ where *c*_*s*_ corresponds to one SD above the mean activity of gene set *s* in all samples. This corresponds to a pattern in which all gene sets are highly active (Figure 3D).

If one chooses ‘Interactive POI’ (Figure 2), the POI will be defined using a computer mouse. For analyzing two gene sets, one can draw one or more polygons on the scatter plot to specify the POI. In the MYC analysis, for instance, MYC expression and its target gene activity are correlated well in most cases except for a small subset of samples where MYC expression is low but the target gene activity is relatively high. To study these samples, a polygon can be drawn as in Figure 3E. The polygon defines the POI. In order to help others to reproduce the results, one can save the interactively specified POI by using ‘Save Current POI’. In a new analysis, one can load previously saved POI using ‘Load POI’.

If one chooses ‘Formulaic POI’ (Figure 2), the POI will be defined using a formula to specify the relationship among gene sets (see Supplementary Materials and Supplementary Figure S2 for details). For example, the formula ‘$(MYC+2)^2+(MYC\_TG-2)^2*10 < 4$’ defines an ellipse (Supplementary Figure S2A). Samples selected by this POI also had low MYC expression and relatively high target gene activity.

Defining POI numerically is simple, and it can be easily incorporated into users’ own analysis pipelines to run analyses automatically. Defining POI interactively, on the other hand, allows one to handle complex POIs. This provides flexibility to explore complex data, but it cannot be automated. Defining POI using formulas is less flexible than using interactive POI but more flexible than using numeric POI, and it may be incorporated into users’ automatic analysis pipelines.

#### Step 4: explore GSCA results

Once the POI is specified, GSCA will search the user-specified compendium and report biological contexts significantly associated with the POI based on user-specified *P*-value and fold change cutoff. The reported contexts along with various summary statistics such as fold change and adjusted *P*-value will be listed in a table and shown under the ‘Ranking Table’ tab (Figure 2). For instance, Figure 3F shows the top biological contexts reported for Figure 3D when the default POI (i.e. high MYC expression and high target gene activity) is used (see Supplementary Table S3 for full results). These contexts are potentially associated with active MYC TF functions. Some contexts were known to have active MYC function (e.g. B cell lymphoma (5)), whereas others were new contexts in which MYC's function was unestablished at the time the 51 core target genes were originally reported. One example for the latter is the Ewing sarcoma (A673 cell line), for which MYC function has been experimentally verified only recently (4). Indeed, 25 of the 30 contexts reported by GSCA (including Ewing tumor) were not used to construct the 51 target gene set (Supplementary Table S3). This provides an example that illustrates how one may use GSCA to link gene sets to previously unknown biological contexts to make new discoveries.

Among all 30 predicted contexts, at least 21 were supported by published experiments (including functional experiments that assess the phenotype changes after perturbing MYC expression or experiments that evaluate MYC protein activity) according to existing literature (Supplementary Table S3). This yields an estimated false discovery rate (FDR) of 30%. This FDR is likely a conservative estimate, since it is possible that some of the remaining 9 predictions are true but have yet to be studied. For this reason, the actual FDR might be lower than 30%. Unlike FDR, evaluating sensitivity is more difficult due to the lack of comprehensive knowledge about the relationship between biological contexts and gene set activities. A series of analyses in Supplementary Materials, Supplementary Figures 3 and 4, and Supplementary Table S3 suggest that the current GSCA can discover a large fraction but not all gene-set-context relationships due to constraints such as sample size. These analyses also show that the continual growth of PED can potentially bring the sensitivity to a high level. Importantly, even though GSCA may not find everything, its reported contexts can already allow one to make many new discoveries (e.g. Ewing tumor in the default POI analysis above), greatly expanding our current knowledge about genes and pathways.

**Figure 4.**
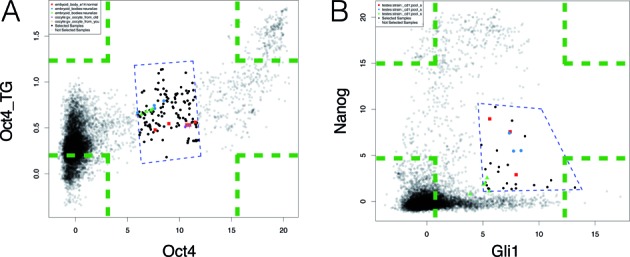
Two examples comparing GSCA interactive POI and ChIP-PED POI. Green dashed lines represent four possible ChIP-PED POIs. Blue dashed lines define the interactive POI in GSCA. (**A**) Analysis of *Oct4* and its target genes (*Oct4*_TG). (**B**) Analysis of *Gli1* and *Nanog*.

After GSCA analysis, samples from the most significant contexts are color-coded and highlighted in the plot shown under the ‘Plot’ tab (Figure 2). For example, Figure 3G shows that samples chosen by the polygon in Figure 3E are highly enriched in Wilms tumor. Since these samples have low MYC expression but relatively high target gene set activity, the plot suggests that MYC target genes may be activated in Wilms tumor by other regulators, or the measurements of MYC RNA level in these samples may not reflect the underlying true MYC protein level.

#### Step 5: save the results

By selecting ‘Save Results’ in the main menu (Figure 2), users can save the list of reported biological contexts to a text file (e.g. Supplementary Table S3). Histograms, scatter plots and heat maps can be exported into PNG, TIFF or other file types for publication. The GUI also provides numerous options for users to customize their plots.

#### Robustness to noises in gene sets

In the above analysis, the MYC target gene set is clean and contains little noise. To see how noises in a gene set may affect GSCA, we replaced 25%, 50%, 75% and 90% of genes in the MYC target gene set by random genes and repeated the analysis using the default POI (Supplementary Materials). This analysis shows that GSCA is relatively robust to noisy genes in the gene sets. Replacing ≤50% of the MYC target genes by noise only resulted in small changes in sensitivity and FDR. The performance decrease was obvious only when >75% of MYC target genes were replaced by noise (Supplementary Figure S5).

### Example II: a comparison with ChIP-PED

The analysis of MYC and its target genes can also be conducted using ChIP-PED. However, ChIP-PED has a number of limitations.

First, ChIP-PED can only analyze two gene sets (a TF and its target genes), whereas GSCA can also analyze one and multiple gene sets (see Examples III and IV below). In addition, GSCA users have the freedom to specify genes’ weights *ω*_*g*_, whereas ChIP-PED users do not. The usefulness of this freedom is demonstrated by an example in Supplementary Materials, Supplementary Figure S6 and Supplementary Table S4, where using a weighting scheme more complex than ±1 improves the analysis.

Second, ChIP-PED only allows one to analyze four possible patterns: (i) high TF and high target gene activity (‘*y*_1*i*_ > *c*_1*H*_ and *y*_2*i*_ > *c*_2*H*_’); (ii) low TF and high target gene activity (‘*y*_1*i*_ < *c*_1*L*_ and *y*_2*i*_ > *c*_2*H*_’), (iii) high TF and low target gene activity (‘*y*_1*i*_ > *c*_1*H*_ and *y*_2*i*_ < *c*_2*L*_’), and (iv) low TF and low target gene activity (‘*y*_1*i*_ < *c*_1*L*_ and *y*_2*i*_ < *c*_2*L*_’). These correspond to the four corners (green dashed lines) in Figure 4. By contrast, GSCA users can interactively specify POIs with arbitrary shapes. This flexibility is crucial in real applications since POIs in real data can be far more complex than those specified by ChIP-PED. For example, Figure 4A shows the TF OCT4 and its target gene activities in mouse (OCT4 target genes were obtained from (4) and listed in Supplementary Table S5 along with their weights). One may ask what sample types are associated with medium levels of *Oct4* and its target gene activity. This question cannot be directly answered using ChIP-PED. However, it can be easily answered using GSCA by drawing a polygon to select samples of interest (Figure 4A, blue dashed lines). It turns out that the selected samples were enriched in differentiating stem cells such as embryoid bodies (Supplementary Table S5). By contrast, samples with high *Oct4* and high target gene activity were enriched in undifferentiated stem cells (Supplementary Figure S7, Supplementary Table S5), consistent with the knowledge that OCT4 protein activity decreases as stem cells differentiate (20). Figure 4B provides another example which analyzes biological contexts associated with *Gli1* and *Nanog* activities in mouse. In this example, which is discussed in detail in Supplementary Materials and Supplementary Figure S8, one POI corresponds to high level of *Gli1* and medium level of *Nanog*. Once again, this can be studied easily using GSCA but not by ChIP-PED. These examples demonstrate that POIs in real application vary substantially from one case to another. It is difficult to have an automatic algorithm smart enough to handle all possible scenarios. Therefore, the versatility provided by GSCA to interactively specify POIs is crucial to meet the diverse needs of different investigators.

**Figure 5.**
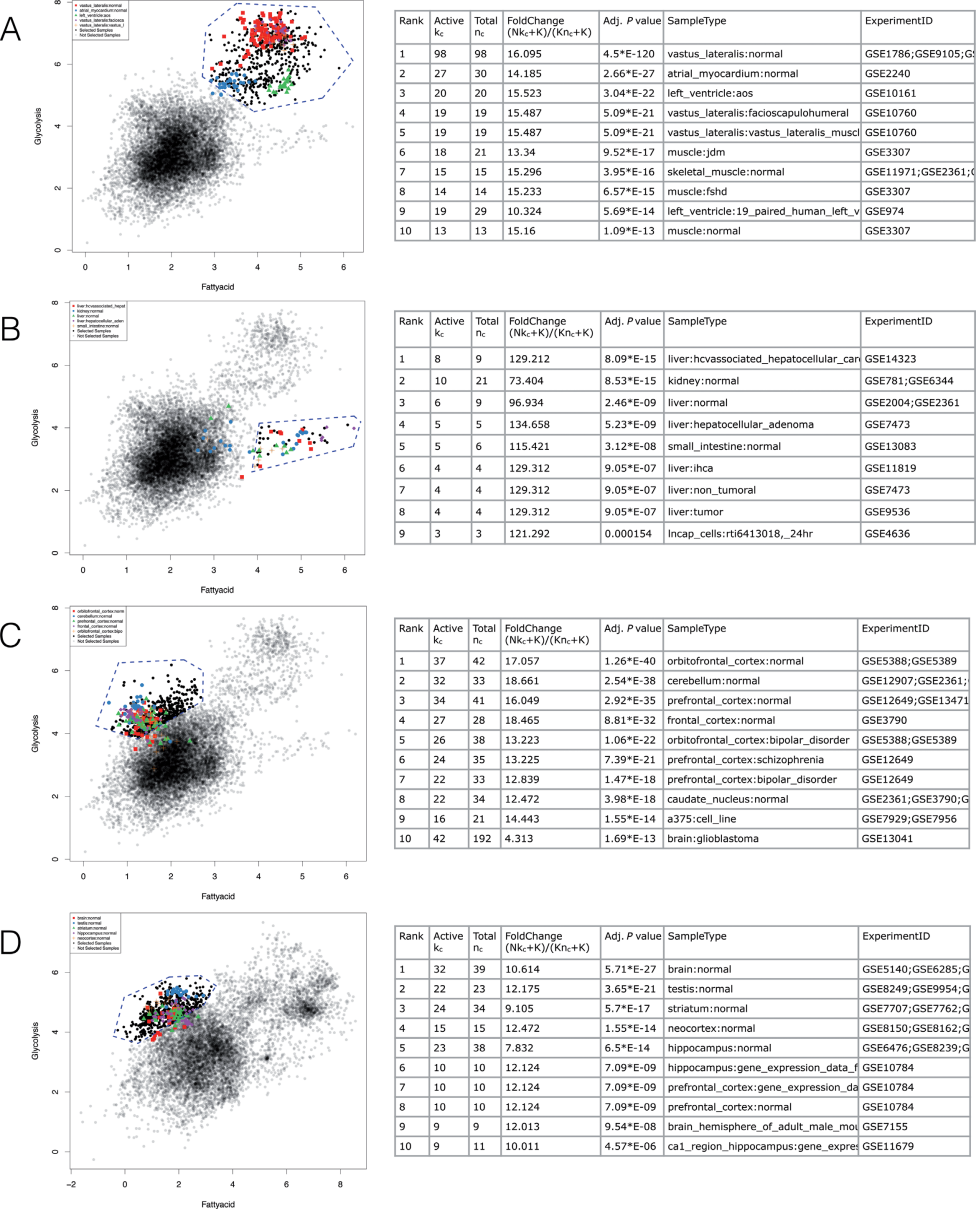
GSCA applied to two metabolic gene sets: glycolysis and fatty acid oxidation. In each panel, blue dashed lines define the interactive POI, and the table shows contexts associated with the POI. (**A**) Analysis of high glycolysis and high fatty acid oxidation in human. (**B**) Medium glycolysis and high fatty acid oxidation in human. (**C**) High glycolysis and medium to low fatty acid oxidation in human. (**D**) High glycolysis and medium to low fatty acid oxidation in mouse.

Third, ChIP-PED requires one input gene set to be a TF and the other gene set to be its target genes. By contrast, GSCA can be used to analyze any gene sets. To demonstrate, Figure 5 shows the GSCA analysis applied to two metabolic pathways, glucose (glycolysis) metabolism and fatty acid oxidation (FAO), in the compendium of Affymetrix Human Genome U133A Array. The two gene sets used for the analysis are provided in Supplementary Table S6. They were obtained from MSigDB (21). Genes shared by both pathways were removed. Genes’ average expression level in each pathway was plotted. Interestingly, samples with high activities of both pathways were highly enriched in muscles (Figure 5A), consistent with the high metabolism rate in muscles (22). There is a group of samples with high fatty acid pathway activity but medium level of glycolysis activity (Figure 5B). These were highly enriched in liver, in which hepatocytes participate in energy utilization and storage and rely on fatty acid oxidation for homeostasis (23). Finally, one can see another group of samples whose glycolysis activity is disproportionally higher than what would be expected by other samples with similar level of fatty acid metabolism (Figure 5C). These samples were highly enriched in brain tissues, which were highly dependent on glucose and glycolysis that prepares glucose catabolites for mitochondrial oxidation (24).

Fourth, ChIP-PED does not provide functions to support cross-species analyses. In GSCA, one can easily convert genes in a gene set to their homologs in another species using HomoloGene (25), and then perform analyses in the converted species. For instance, we converted the glycolysis and fatty acid oxidation gene sets from human to mouse using ENTREZ ID conversion tool included in GSCA GUI. We then run GSCA in mouse. Figure 5D shows an analysis similar to Figure 5C. Samples with glycolysis activity higher than expected given the level of fatty acid metabolism were again enriched in brain tissues. Thus, the finding related to this pattern is conserved between human and mouse.

In addition to the differences above, GSCA also provides numerous functions to help users conveniently tune parameters, save interactively defined POIs, and export analysis results such as plots and tables. These functions are not available in ChIP-PED.

### Example III: analysis of one gene set

**Figure 6.**
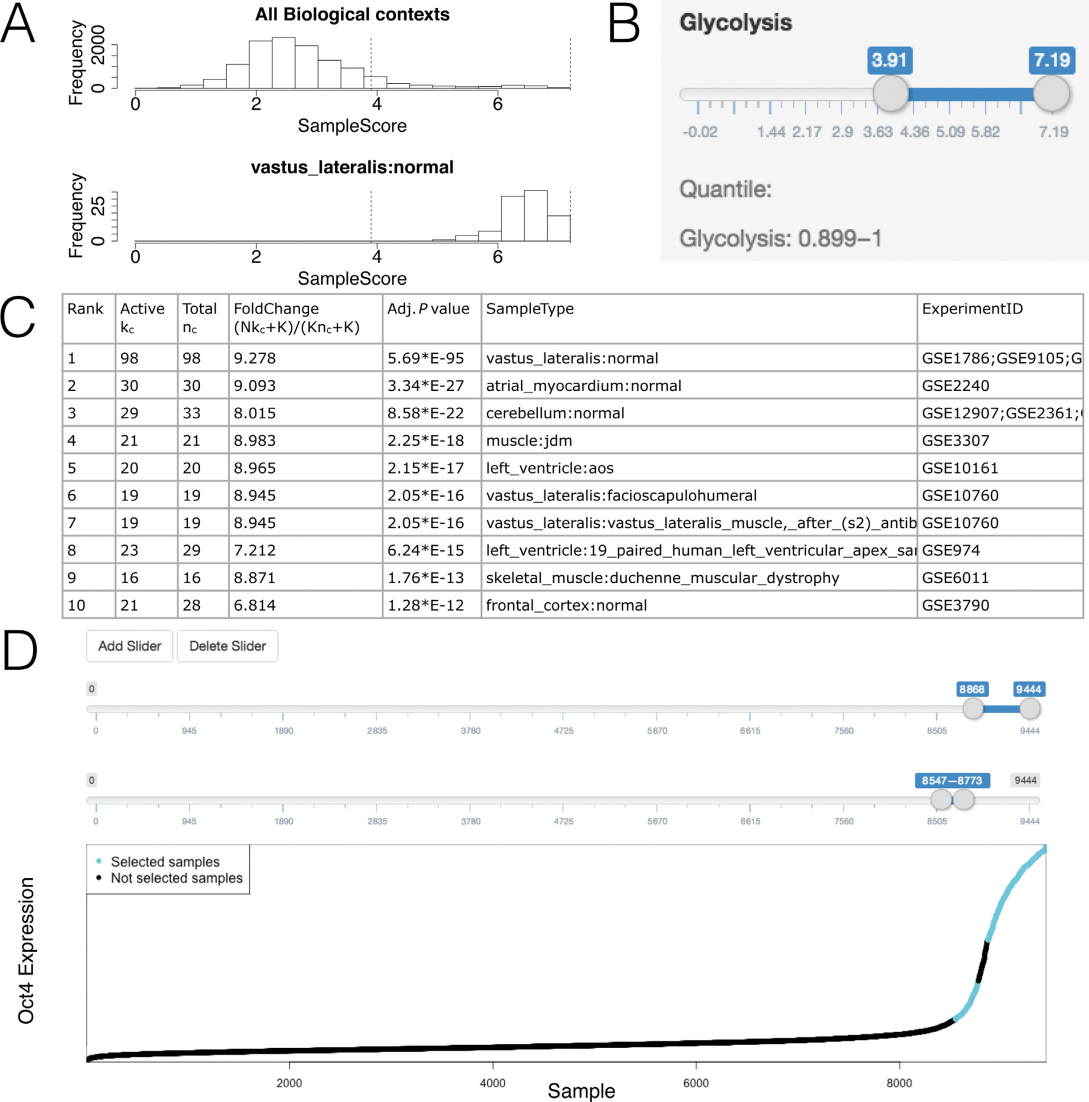
GSCA for one gene set. (**A**) Histograms showing gene set activities across samples. (**B**) Sliders used to define POI. (**C**) Table showing the identified biological contexts. (**D**) Complex POI can be defined as the union of multiple intervals, specified interactively using multiple slider bars.

Unlike ChIP-PED which can only analyze two gene sets, GSCA can also analyze one or multiple gene sets. Figure 6 demonstrates GSCA for one gene set. GSCA was used to analyze the glycolysis gene set in Supplementary Table S6 using the compendium of Affymetrix Human Genome U133A Array. GSCA generates a number of histograms (Figure 6A) showing the distributions of gene set activity in all samples as well as in samples from the top significant biological contexts associated with the default POI (i.e. high glycolysis activity). Users can now specify their own POI numerically by using either a keyboard or a slider. For example, in Figure 6B, the slider was set to choose samples whose glycolysis activity is above the 90th percentile of all samples. The GSCA results show that these samples were highly enriched in muscles and brain tissues (Figure 6C). One can also specify POI interactively by using multiple sliders to select multiple intervals whose union defines the POI (Figure 6D). This is demonstrated in more detail by an analysis of *Oct4* in Supplementary Materials and Supplementary Figure S9. Defining POI using formulas is also supported. This is similar to how formulas were used in Example I and therefore a detailed discussion is skipped here for brevity.

### Example IV: analysis of multiple gene sets

To demonstrate GSCA for multiple gene sets, we analyzed MYC and three metabolic pathways—mitochondria biogenesis, glycolysis and fatty acid oxidation—in the compendium of Affymetrix Human Genome U133A Array (see Supplementary Table S6 for gene sets, *ω*_*g*_ = 1). The three metabolic gene sets were obtained from MSigDB (21) and then filtered to exclude genes that are not MYC target genes. To do so, we first compiled a list of MYC binding sites by taking the union of signal peaks using available MYC ChIP-seq data from ENCODE (hg19) listed in Supplementary Table S7. A gene was retained for analysis if there was at least one MYC binding site within the 5 kb upstream and 1 kb downstream of the gene's transcription start site. We further filtered genes so that no genes were shared by any two gene sets. Previous research has shown that the MYC oncogene is involved in amplifying the expression of genes involved in metabolism, cell growth and cell death (26). Here, we sought to identify the biological contexts in which MYC might affect different metabolic pathways by directly or indirectly regulating metabolic genes.

**Figure 7.**
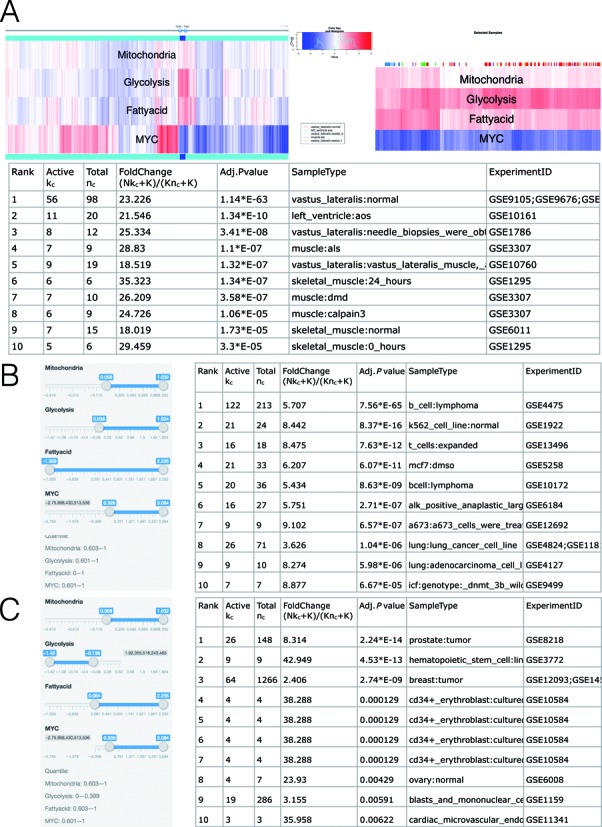
GSCA for multiple gene sets. (**A**) Gene set activities across all PED samples are shown using a heat map (left). The sliders above the heat map can be used to interactively define POI. The heat map on the right provides a zoom-in view of the slider selected samples. The table below shows the identified biological contexts. (**B**) POI can also be defined by setting a numeric range for each gene set using sliders. This panel shows an analysis of high MYC, high mitochondria and high glycolysis activities. (**C**) High MYC, high mitochondria, high fatty acid and low glycolysis.

After providing the gene sets and PED compendium, GSCA creates a heat map showing activities of the four gene sets in all PED samples (Figure 7A, left). In the heat map, rows represent gene sets, columns represent samples, colors represent gene set activities, and samples are clustered using hierarchical clustering (Euclidean distance measure, complete linkage method). Users have the option to cluster gene sets, or to adjust the display order of the gene sets in the heat map using the GUI.

Users can now specify their own POI interactively by using the slider above the heat map to select samples of interest. One may add multiple sliders to define multiple intervals and use their union to define POI. In Figure 7A, there is a group of samples with low MYC expression but high mitochondrial, glycolytic and fatty acid gene activities. After users have selected these samples and clicked ‘Update Sample Selection’ button, GSCA creates a second heat map to provide a zoom-in view of the selected samples (Figure 7A, right). One can find that these samples are enriched in various types of muscles (Figure 7A, bottom). This low MYC high metabolic expression signature is consistent with the high metabolic rates of the non-proliferating muscle tissues.

POIs can also be defined numerically. One can either type numerical cutoffs in text boxes or use sliders to set a numeric range for each gene set. In Figure 7B, for instance, we used sliders to select samples with high MYC expression and high expression of mitochondrial and glycolysis genes (‘high’ was defined approximately using the 60th percentile of the expression of each gene set across all samples). Biological contexts associated with this pattern include a variety of cancer cell lines such as B cell lymphoma and K562. MYC has been implicated to drive the expression of glycolytic genes in cancer cells (26), thus the GSCA finding of these cancer cell lines for high MYC and high glycolysis is consistent with existing literature. Interestingly, GSCA revealed that these cancer cells have concurrently high mitochondrial and glycolytic signatures, suggesting that they use both pathways in their highly proliferative state. This is a previously under-appreciated phenomenon.

Further analyses show that samples with high MYC, high mitochondria and fatty acid, but low glycolysis activity (‘low’ was defined approximately using the 40th percentile of the expression of each gene set across all samples) were enriched in hematopoietic stem cells and CD34+ cultured erythroblasts, as well as breast and prostate cancer tissues (Figure 7C). This is intriguing since unlike the widely-recognized role of MYC in glucose metabolism, mitochondrial biogenesis, and lipogenesis, its role in fatty acid oxidation or degradation by mitochondria is less-well understood (27). On the one hand, cells stimulated to grow by MYC relies on glucose and glutamine for biosynthesis of macromolecules such as fatty acids through lipogenesis, which has a feed-back loop through malonyl-CoA to inhibit mitochondrial fatty acid oxidation. On the other hand, MYC was shown experimentally to induce fatty acid oxidation through mitochondrial biogenesis, such that shutting down MYC expression resulted in accumulation of intracellular lipid droplets in tumor cells (28). Hence, there is a gap of knowledge regarding the role of MYC in FAO. The use of GSCA may identify biological contexts that reveal tissue specific alterations of FAO by MYC. In this regard, Figure 7C shows contexts with high MYC, high mitochondria and high FAO gene expression, including breast and prostate cancer tissues, hematopoietic stem cells among other contexts. The possibility that hematopoietic stem cells (CD34+) may use fatty acid oxidation is implicated in the literature (29). Several studies also suggest that leukemia hematopoietic stem cells (CD34+) are sensitive to fatty acid oxidation inhibition with etomoxir (30). These provide support for the GSCA findings. Interestingly, the hematopoietic stem cell and CD34+ samples were not enriched in high MYC-expressing samples in which only one of the mitochondria and fatty acid gene sets was active (Supplementary Figure S10), indicating that both metabolic pathways are important for hematopoietic stem cell and CD34+ cells. These observations provide clues for further experimentation to determine the tissue and cell type role of MYC in influencing FAO and may result in new insights for cancer therapy.

Finally, one can also define POI using formulas. For instance, Supplementary Figure S11 illustrates how an analysis similar to Figure 7A can be done by directly typing a formula.

## DISCUSSION

In summary, we have demonstrated how GSCA can be used to explore gene set activities in a large compendium of PED samples and link interesting gene set activity patterns to biological contexts. In our examples, GSCA revealed both known and unknown contexts. The new contexts may provide an informative guide to help people design future experimental studies to investigate new and previously unsuspected biology. Potentially, GSCA may also be used as a hypothesis screening tool to quickly explore different hypotheses in order to pick up the most promising ones for designing follow-up studies. Today's high-throughput experiments often produce new gene sets as products. Although such experiments are powerful, most investigators only have resources for a limited number of such experiments. For these investigators, GSCA provides a cost-efficient and readily available way to boost the value of their data and make new discoveries. With the GUI and ability to interactively specify POI, GSCA can be used conveniently in one's daily research.

GSCA currently uses Fisher's exact test to determine the statistical significance of biological contexts. This test is based on assuming that samples are independent. Adjusted *P*-values produced by GSCA therefore should be interpreted with respect to this assumption. Empirically, we found that this approach can produce reasonable results as demonstrated by our examples. Previously, methods developed for handling correlation among genes in the conventional gene set analysis, such as ROAST (31) and CAMERA (32), have been found useful for improving statistical inference. It will be interesting to investigate in the future whether GSCA can be generalized in a similar fashion to allow potential correlation among samples and whether such generalization can produce better analysis results. As further discussed in Supplementary Materials and Supplementary Figure S12, handling correlation among samples in GSCA is not easy as it requires one to address non-trivial issues such as considering inter-gene and inter-sample correlations at the same time and estimating complex high-dimensional correlation structure among samples. When using the adjusted *P*-values in the GSCA output, users should also keep in mind that these *P*-values measure statistical significance only when GSCA is used to answer a specific question involving a specific POI defined before looking at the GSCA results (see Supplementary Materials). If users do not have a specific question in mind and want to repeatedly explore different POIs (e.g. by interactively tuning a POI based on GSCA results) until something ‘significant’ is reported, these *P*-values can no longer be used as a formal statistical significance measure since they were not designed to capture the uncertainty associated with such data snooping. In this scenario, GSCA is purely exploratory in nature, and *P*-values may only be used along with other statistics (e.g. fold changes) to rank biological contexts rather than telling the probability that they were reported by chance. Users should use other independent sources of information to verify the validity of the ‘findings’. How to measure statistical significance in an exploratory analysis involving human-machine interactions remains an open problem that is worth future investigation.

There are a number of other potential directions to extend GSCA. First, the current GSCA relies on microarray samples curated by BARCODE. As the normalization and curation of RNA-seq data mature, one may extend GSCA to incorporate RNA-seq compendia. Second, we currently analyze each PED compendium separately. Integrative models for multiple-platform and cross-species GSCA are worth future investigation. Third, GSCA requires one to have annotated samples. Currently, samples in BARCODE are manually annotated and curated. In order to take the full advantage of the fast growing data, developing an automatic annotation system using the GEO metadata and incorporating it with GSCA is crucial.

## SUPPLEMENTARY DATA

Supplementary Data are available at NAR online.

SUPPLEMENTARY DATA

## References

[1] Edgar R., Domrachev M., Lash A.E. (2002). Gene expression omnibus: Ncbi gene expression and hybridization array data repository. Nucleic Acids Res..

[2] Brazma A., Parkinson H., Sarkans U., Shojatalab M., Vilo J., Abeygunawardena N., Holloway E., Kapushesky M., Kemmeren P., Lara G.G. (2003). ArrayExpress–a public repository for microarray gene expression data at the EBI. Nucleic Acids Res..

[3] Subramanian A., Tamayo P., Mootha V.K., Mukherjee S., Ebert B.L., Gillette M.A., Paulovich A., Pomeroy S.L., Golub T.R., Lander E.S. (2005). Gene set enrichment analysis: a knowledge-based approach for interpreting genome-wide expression profiles. Proc. Natl. Acad. Sci. U.S.A..

[4] Wu G., Yustein J.T., McCall M.N., Zilliox M., Irizarry R.A., Zeller K., Dang C.V., Ji H. (2013). ChIP-PED enhances the analysis of ChIP-seq and ChIP-chip data. Bioinformatics..

[5] Ji H., Wu G., Zhan X., Nolan A., Koh C., De Marzo A., Doan H.M., Fan J., Cheadle C., Fallahi M. (2011). Cell-type independent MYC target genes reveal a primordial signature involved in biomass accumulation. PLoS One..

[6] Zilliox M.J., Irizarry R.A. (2007). A gene expression bar code for microarray data. Nat. Methods..

[7] Hibbs M.A., Hess D.C., Myers C.L., Huttenhower C., Li K., Troyanskaya O.G. (2007). Exploring the functional landscape of gene expression: directed search of large microarray compendia. Bioinformatics..

[8] Greene C.S., Troyanskaya O.G. (2011). PILGRM: an interactive data-driven discovery platform for expert biologists. Nucleic Acids Res..

[9] Butte A.J., Kohane I.S. (2006). Creation and implications of a phenome-genome network. Nat. Biotechnol..

[10] Huttenhower C., Haley E.M., Hibbs M.A., Dumeaux V., Barrett D.R., Coller H.A., Troyanskaya O.G. (2009). Exploring the human genome with functional maps. Genome Res..

[11] Xu M., Li W., James G.M., Mehan M.R., Zhou X.J. (2009). Automated multidimensional phenotypic profiling using large public microarray repositories. Proc. Natl. Acad. Sci. U.S.A..

[12] Huang H., Liu C.C., Zhou X.J. (2010). Bayesian approach to transforming public gene expression repositories into disease diagnosis databases. Proc. Natl. Acad. Sci. U.S.A..

[13] Lukk M., Kapushesky M., NikkilÁ J., Parkinson H., Goncalves A., Huber W., Ukkonen E., Brazma A. (2010). A global map of human gene expression. Nat. Biotechnol..

[14] Wu G., Ji H. (2013). ChIPXpress: using publicly available gene expression data to improve ChIP-seq and ChIP-chip target gene ranking. BMC Bioinformatics..

[15] Petryszak R., Burdett T., Fiorelli B., Fonseca N.A., Gonzalez-Porta M., Hastings E., Huber W., Jupp S., Keays M., Kryvych N. (2014). Expression Atlas update–a database of gene and transcript expression from microarray-and sequencing-based functional genomics experiments. Nucleic Acids Res..

[16] McCall M.N., Jaffee H.A., Zelisko S.J., Sinha N., Hooiveld G., Irizarry R.A., Zilliox M.J. (2014). The Gene Expression Barcode 3.0: improved data processing and mining tools. Nucleic Acids Res..

[17] McCall M.N., Bolstad B.M., Irizarry R.A. (2010). Frozen robust multiarray analysis (fRMA). Biostatistics..

[18] Altschul S.F., Gish W., Miller W., Myers E.W., Lipman D.J. (1990). Basic local alignment search tool. J. Mol. Biol..

[19] Fisher R.A. (1915). Frequency distribution of the values of the correlation coefficient in samples from an indefinitely large population. Biometrika..

[20] Niwa H., Miyazaki J.I., Smith A.G. (2000). Quantitative expression of Oct-3/4 defines differentiation, dedifferentiation or self-renewal of ES cells. Nat. Genet..

[21] Liberzon A., Subramanian A., Pinchback R., Thorvaldsdóttir H., Tamayo P., Mesirov J.P. (2011). Molecular signatures database (MSigDB) 3.0. Bioinformatics..

[22] Horscroft J.A., Murray A.J. (2014). Skeletal muscle energy metabolism in environmental hypoxia: climbing towards consensus. Extrem. Physiol. Med..

[23] Rui L. (2014). Energy Metabolism in the Liver. Compr. Physiol..

[24] Schurr A. (2014). Cerebral glycolysis: a century of persistent misunderstanding and misconception. Front Neurosci..

[25] Geer L.Y., Marchler-Bauer A., Geer R.C., Han L., He J., He S., Liu C., Shi W., Bryant S.H. (2009). The NCBI biosystems database. Nucleic Acids Res..

[26] Dang C.V. (2013). MYC, metabolism, cell growth, and tumorigenesis. Cold Spring Harb. Perspect. Med..

[27] Dang C.V. (2012). MYC on the Path to Cancer. Cell.

[28] Zirath H., Frenzel A., Oliynyk G., Segerström L., Westermark U.K., Larsson K., Persson M.M., Hultenby K., Lehtiö J., Einvik C. (2013). MYC inhibition induces metabolic changes leading to accumulation of lipid droplets in tumor cells. Proc. Natl. Acad. Sci. U.S.A..

[29] Ito K., Carracedo A., Weiss D., Arai F., Ala U., Avigan D.E., Schafer Z.T., Evans R.M., Suda T., Lee C.H. (2012). A PML-PPAR-δ pathway for fatty acid oxidation regulates hematopoietic stem cell maintenance. Nat. Med..

[30] Samudio I., Harmancey R., Fiegl M., Kantarjian H., Konopleva M., Korchin B., Kaluarachchi K., Bornmann W., Duvvuri S., Taegtmeyer H. (2010). Pharmacologic inhibition of fatty acid oxidation sensitizes human leukemia cells to apoptosis induction. J. Clin. Invest..

[31] Wu D., Lim E., Vaillant F., Asselin-Labat M., Visvader J. E., Smyth G.K. (2010). ROAST: rotation gene set tests for complex microarray experiments. Bioinformatics..

[32] Wu D., Smyth G.K. (2012). Camera: a competitive gene set test accounting for inter-gene correlation. Nucleic Acids Res..

