# Supplementary material for: Turning publicly available gene expression data into discoveries using gene set context analysis
Source: Nucleic Acids Res. 2015 Sep 8;44(1):e8. doi: 10.1093/nar/gkv873 (PMC4705686; doi:10.1093/nar/gkv873)
Supplement: SUPPLEMENTARY DATA [file supp_44_1_e8__index.html]

Turning publicly available gene expression data into discoveries using gene set context analysis — SUPPLEMENTARY DATA 

# Turning publicly available gene expression data into discoveries using gene set context analysis

## SUPPLEMENTARY DATA

- SUPPLEMENTARY DATA
- SUPPLEMENTARY DATA
- SUPPLEMENTARY DATA
- SUPPLEMENTARY DATA
- SUPPLEMENTARY DATA
- SUPPLEMENTARY DATA
- SUPPLEMENTARY DATA
- SUPPLEMENTARY DATA
