# Supplementary material for: Turning publicly available gene expression data into discoveries using gene set context analysis
Source: Nucleic Acids Res. 2015 Sep 8;44(1):e8. doi: 10.1093/nar/gkv873 (PMC4705686; doi:10.1093/nar/gkv873)
Supplement: SUPPLEMENTARY DATA [file supp_gkv873_nar-00555-met-z-2015-File002.pdf]

Supplementary Materials

Contents

Supplementary Methods and Results

1. Sample Annotation
   Annotation of biological contexts
   Annotation comprehensiveness
2. Positive and Negative Weights for Defining Gene Set Activity
3. Installation of GSCA Software
4. Defining POI Using Formulas
5. Analysis of Sensitivity
6. Impact of Noisy Gene Set
7. Demonstration of Non-integer Weights
8. GSCA Analysis of *Gli1* and *Nanog*
9. GSCA Analysis of *Oct4*
10. Caveats in Statistical Inference
    Interpretation of statistical significance in interactive analysis
    Correlations among samples

List of Supplementary Tables

- Supplementary Table 1 (xlsx format): Evaluation of annotation comprehensiveness. A list of 30 major tissue types and a list of 167 major cell types in mammals are compiled. The table has multiple sheets that show whether each tissue or cell type is covered by our PED annotation or by GEO metadata.
- Supplementary Table 2 (csv format): A table that lists MYC and its 51 core target genes used for Example I. The table can be directly uploaded to GSCA GUI.

- Supplementary Table 3 (xlsx format): The table contains multiple sheets that show GSCA results for Example I (MYC and target genes). "Active" shows k_c; "Total" shows n_c; "Fold Change" shows f_c. The table also lists the 16 MYC-related contexts and whether they are significant, in POI or in the compendium.

- Supplementary Table 4 (xlsx format): A table with multiple sheets that lists the gene set and gene weights used for comparing the simple and complex weighting schemes. The GSCA results for the two weighting schemes are also listed. Contexts discussed in the Supplementary Materials are highlighted with red in complex weighting scheme.

- Supplementary Table 5 (csv format): A table with multiple sheets that lists (1) *Oct4* and its target gene set used for Example II (The table can be directly uploaded to GSCA GUI), and (2) GSCA analysis of *Oct4* and its target genes.

- Supplementary Table 6 (csv format): A table with multiple sheets that lists (1) the glycolysis gene set used for Example III, (2) the glycolysis and fatty acid oxidation gene sets used for Example II, and (3) MYC, mitochondria biogenesis, glycolysis, and fatty acid oxidation gene sets used for Example IV. All sheets can be directly uploaded to GSCA GUI. For sheet 3, since most FAO genes were also included in the mitochondria gene set, we first removed genes from the mitochondria gene set if they belonged to the FAO gene set. From the resulting gene sets, we then removed overlapping genes between any pair of gene sets.

- Supplementary Table 7 (csv format): A table that lists all the web links where MYC ChIP-seq peak files are downloaded.

List of Supplementary Figures

- Supplementary Figure 1: Illustration of using positive and negative weights in computing gene set activity. A: The expression of *E2f7* (y-axis) is positively correlated with *Myc* expression (x-axis). The Pearson correlation coefficient is shown above the plot. B: *Pink1* is negatively correlated with *Myc*. C: When the weight for *E2f7* is 1 and the weight for *Pink1* is -1, the gene set activity defined using the weighted average of *E2f7* and *Pink1* expression is positively correlated with *Myc*, and the correlation is stronger than (A) and (B). D: When the weights for *E2f7* and *Pink1* are both equal to 1, the gene set activity is equal to the average expression of *E2f7* and *Pink1*. It is not strongly correlated with *Myc*.

- Supplementary Figure 2: Illustration of defining POI using formulas. A: Analysis of MYC and its 51 core target genes in Example I. The POI is defined using formula "$(MYC+2)^2+(MYC\_TG-2)^2*10 < 4$". Samples in the POI region are highlighted by dark black. B: Top enriched biological contexts in (A) and all their samples are shown in color. C: The top two

enriched biological contexts in (A) are both related to Wilms tumor. D: Analysis of glycolysis and fatty acid oxidation gene sets in Example II. The POI is defined using formula "Glycolysis $<$ Fattyacid & Fattyacid $>$ 4". Samples in the POI region are highlighted by dark black. E: Top enriched biological contexts in (D) and all their samples are shown in color. F: The top enriched biological contexts in (D) are liver.

- Supplementary Figure 3: Sample size and fold change distribution of five different types of biological contexts. A: Violin plots showing the distribution of $\log_2(sample\ size)$ (i.e., $\log_2(n_c)$). B: Violin plots showing the distribution of $\log_2(fold\ change)$ (i.e., $\log_2(f_c)$). From left to right, the five biological context types are (1) contexts appearing in the POI region that passed the significance cutoff of GSCA, (2) contexts appearing in the POI region that did not pass the GSCA significance cutoff, (3) the 7 gold standard MYC contexts appearing in the POI region that passed the GSCA significance cutoff, (4) the 6 gold standard MYC contexts appearing in the POI region that did not pass the GSCA significance cutoff, (5) all contexts in the PED compendium.

- Supplementary Figure 4: Sensitivity and FDR of GSCA in simulations with increasing sample size. In the simulation study, the sample size per context is increased by multiplying a factor α, and the number of context is increased by multiplying another factor β. The total sample count in the compendium is approximately $\alpha\beta N$ after simulation where N is the original total sample count. For each (α, β), the simulation was run five times, and the average performance of these five runs is shown. A: Sensitivities for detecting the 16 gold standard MYC contexts in the original MYC analysis (Example I, default POI) and four simulations with different (α, β). B: FDR.

- Supplementary Figure 5: GSCA performance in the MYC analysis (Example I, default POI) after replacing certain percentage (0%, 25%, 50%, 75%, 90%) of the 51 MYC target genes with noise (i.e., randomly chosen genes). A: The number of significant contexts reported by GSCA at its default cutoff. B: FDR. C: Sensitivity for detecting the 16 gold standard MYC contexts. For each noise percentage, the noise replacement process was repeated 5 times independently. The plots show the mean and range of the five runs.

- Supplementary Figure 6: GSCA analysis of the SHH gene set with the default POI using two different weighting schemes. A: Simple weighting scheme where all genes have equal weights. B: Complex weighting scheme where genes' weights depend on their moderated t-statistics.

- Supplementary Figure 7: GSCA analysis of *Oct4* and its target genes. A: In the POI selected samples, *Oct4* and its target gene activities are both high. B: *Oct4* and its target gene activities are both at medium level.

Plots on the left show the POIs. Tables on the right show the enriched biological contexts.

- Supplementary Figure 8: GSCA analysis of *Gli1* and *Nanog*. A: *Gli1* and *Nanog* are both highly expressed. B: *Gli1* is highly expressed and *Nanog* is expressed at medium level. C: *Gli1* is highly expressed and *Nanog* is lowly expressed.

- Supplementary Figure 9: GSCA for one gene set illustrated using *Oct4*. A: Complex POI can be defined as the union of multiple intervals, specified interactively using multiple slider bars. B: The top 10 enriched biological contexts associated with the chosen POI.

- Supplementary Figure 10: GSCA for multiple gene sets. A: High MYC, high mitochondria, low fatty acid, and low glycolysis. B: High MYC, low mitochondria, high fatty acid, and low glycolysis.

- Supplementary Figure 11: Using formula to define POI in the analysis of MYC and three metabolic pathways (Example IV). The analysis in Figure 7A is similar to using formula-defined POI "$MYC < quantile(MYC, 0.1)$ & $Mitochondria > quantile(Mitochondria, 0.9)$ & $Glycolysis > quantile(Glycolysis, 0.9)$ & $Fattyacid > quantile(Fattyacid, 0.9)$". Here $quantile()$ is the R function for computing quantiles. For example, $quantile(s, 0.9)$ means 90th percentile of the activity of gene set s in all samples. A: Screenshot for the GUI when specifying POI using formula. B: Text box for inputting the formula. C: The enriched biological contexts.

- Supplementary Figure 12: Illustration of permuting genes. A: Original gene set activities of glycolysis and fatty acid gene sets. B-D: Gene set activities after permuting genes. Three independent permutations are shown.

1 Sample Annotation

1.1 Annotation of biological contexts

The annotations of biological contexts used by GSCA is based on the sample annotations provided by BARCODE [1]. The detailed annotation procedure has been described previously in [1, 2]. Briefly, in order to annotate the biological context associated with a sample, the metadata text annotation files (i.e., files named as GSM* and GSE*) linked to the sample were downloaded from GEO. These text documents were read by an expert biologist. Based on information extracted from multiple fields in these files, including "Title", "Source name", "Characteristics", "Description" and "Protocol", the expert determined the cell or tissue type of each sample, and the treatment or disease conditions under which the sample was collected. The biological context of the sample is then defined and annotated as the sample's cell or tissue type and its associated treatment or disease condition. For example, if a sample is obtained by treating MCF7 cells with E2, then the biological context of the sample would be annotated as "MCF7 cells: treated with E2". Here the colon, ":", separates the cell or tissue type from the treatment or disease condition.

In our current annotation system, all biological contexts are disjoint. In other words, each sample is only annotated with one biological context. Although not currently available in GSCA, one could in principle annotate each sample with multiple keywords, treating each keyword as a biological context. For example, a sample can be annotated with "stem cell", "embryonic stem cell" and "undifferentiated" simultaneously, and each of these keywords defines a biological context. In this way, each sample can belong to multiple biological contexts. The keywords can be grouped into different categories (e.g., "male" and "female" are two keywords for "gender"; "stem cell" and "neuron" are two keywords for "cell type"), and there may be internal structures among keywords (e.g., "embryonic stem cell" belongs to "stem cell"). If such a keyword-based annotation is available, one can generalize the current GSCA to test the association of a POI with each keyword. This may make the analysis more flexible. Unfortunately, a structured annotation system based on keywords is still not available for the BARCODE data. Creating such annotations is non-trivial. It requires one to compile and curate an ontology of keywords and analyze GEO annotation documents by text mining. Because the GEO annotation texts are very noisy (e.g., there are many typos and non-standard use of terminology), a sample annotation with good quality also requires one to combine text mining with machine learning and human expert curation. Currently, we are working on building such a system. However, it will take time before this system is fully tested and becomes mature. For this reason, GSCA currently does not provide such an annotation system which we plan to incorporate in the future when it matures and is systematically evaluated.

1.2 Annotation comprehensiveness

To see whether the major tissues and cell types are covered by our annotations, we compiled a list of 30 major tissue types and a list of 167 major cell types by manually integrating information from the TiGER database [3], ENCODE [4] and human expert knowledge (Supplementary Table 1). We checked whether these contexts were covered by our sample annotations. It turns out that the majority of these tissues and cell types was found in our annotations (Supplementary Table 1). Specifically, among the 30 tissue types, 28 (93.3%) were covered by our human PED annotations, 28 (93.3%) were covered by our mouse PED annotations, and 29 (96.7%) were covered by either the human or mouse annotations. Among the 167 cell types, 119 (71.3%) were covered by our human PED annotations, 113 (67.7%) were covered by our mouse PED annotations, and 137 (82.0%) were covered by either human or mouse. These results indicate that the annotations used by GSCA provide a good coverage of the major tissues and cell types.

For tissues and cell types that were not found in our annotations, we asked why they were missing. In particular, we were interested to know whether they were missing because of the lack of corresponding samples in our PED compendium, or whether they were missing only because our sample annotation was not comprehensive enough to cover these terms even though samples from these tissues and cell types were available in our PED compendium. To answer this question, we downloaded GEO metadata for all samples in our PED compendium. The metadata contained detailed text descriptions about each sample (i.e., texts in GEO GSE* and GSM* files that describe sample origins, experimental procedures, etc.). The information in these text documents is more comprehensive than the BARCODE annotation, because the original BARCODE annotation was extracted from these raw texts by a human expert. Using these text documents, we evaluated whether the BARCODE annotation was able to capture the key information in the raw metadata. To do this, we searched the tissue and cell type names in the downloaded text documents. The results are summarized in Supplementary Table 1. It turns out that all 30 tissue types were found in the raw human metadata, and 28 (93.3% of 30) of them were covered by the BARCODE annotation. For mouse, 28 (93.3%) of the 30 tissue names were found in the raw metadata, and all these 28 (100% of 28) were covered by the BARCODE annotation. For the 167 cell types, 120 (71.9% of 167) were found in the raw human metadata. Among them, 119 (99.2% of 120) were covered by the BARCODE annotation. Similarly, 114 (68.3% of 167) cell type names were found in the raw mouse metadata. Among them, 113 (99.1% of 114) were covered by the BARCODE annotation. Thus, most tissue and cell type names present in the raw metadata were captured by the BARCODE annotation. These results indicate that the tissues and cell types not found in the BARCODE annotation were missing mainly because relevant samples were not available in our PED compendium, and the annotation procedure itself was of good quality and was able to capture key context information available in the raw GEO metadata.

We note that in principle gene expression data for each tissue or cell type could be collected under many different conditions (e.g., at different time points, under different treatments, etc.). The combinations of different factors such as tissue/cell type, time, and treatment, etc. can easily create an unlimited number of biological contexts. It is unrealistic to expect our PED compendium and annotation to cover all of these contexts since not all of them have been studied by the scientific community. On the other hand, GSCA is under continuous development. As new data become available, we expect that our PED compendium and annotation will become more comprehensive. Importantly, even though our current PED compendium and annotation may not cover all biological contexts, they do cover a broad spectrum of tissues and cell types which can provide a reasonably good view of the global gene expression landscape in human and mouse. As demonstrated by the original BARCODE study [1], samples from these diverse contexts span both ends of the expression spectrum (i.e., high and low expression) for most genes, and therefore one can reliably tell whether a gene's expression in a particular sample is high or low by comparing it to the gene's expression in all other samples. Our examples in this article further demonstrate that GSCA is capable of recovering known relationships between gene sets and biological contexts (e.g., *Oct4* – embryonic stem cell, MYC – B cell lymphoma). These results confirm that, given the diversity of the currently available samples, incompleteness of our PED compendium and annotation is not a major concern for users to determine the relative high or low activity of a gene set, define POI accordingly, and subsequently identify contexts available in our PED compendium that are associated with the POI.

Clearly, GSCA will not be able to report any biological context that is not present in our PED compendium. Therefore, interpreting GSCA results as "all" biological contexts associated with a POI will be incorrect and misleading. Users should avoid interpreting data in such a way. However, for the purpose of exploring available data and using them to discover new relationships between gene set activities and biological contexts (rather than finding all such relationships), GSCA provides a valuable tool for the community. Our examples (e.g., MYC – Ewing tumor relationship discussed in Example I) show that even with the data and annotation currently available in our compendium, one can make new discoveries and learn many new things. Without GSCA, using PED to do a similar analysis would be difficult.

2 Positive and Negative Weights for Defining Gene Set Activity

We use a simple example to illustrate the use of positive and negative weights to integrate anti-correlated genes. Consider the problem of using target genes of a transcription factor (TF) to study the TF regulatory activity. *E2f7* and *Pink1* are two target genes of transcription factor MYC. It is known that *E2f7* is activated whereas *Pink1* is repressed by MYC in murine T-lymphoma cell

[5]. The scatter plot in Supplementary Figure 1A shows that the expression of *E2f7* is positively correlated with *Myc* expression in our Affymetrix Mouse Genome 430 2.0 Array (GPL1261) compendium (Pearson correlation = 0.487). In the scatter plot, each dot represents a sample in the compendium. Similarly, Supplementary Figure 1B shows that the expression of *Pink1* is negatively correlated with *Myc* expression (Pearson correlation = −0.440). When we combine *E2f7* and *Pink1* to create a target gene set of MYC and assign weight $w_g = 1$ to *E2f7* and $w_g = -1$ to *Pink1*, the gene set activity computed using formula (1) in the main article has higher correlation with the *Myc* expression (Pearson correlation = 0.527) and hence better predicts MYC transcription factor activity than each individual target gene (Supplementary Figure 1C). By contrast, if we use $w_g = 1$ for both *E2f7* and *Pink1*, the resulting gene set activity (i.e., the mean expression of *E2f7* and *Pink1*) does not correlate with *Myc* expression very well (Supplementary Figure 1D, Pearson correlation = 0.088). This shows how the positive and negative weights provide a natural way to integrate information from anti-correlated genes into a single consolidated measure that may be used to conveniently study certain biological questions of interest, such as comparing TF regulatory activities across samples.

3 Installation of GSCA software

GSCA and its supporting data packages are distributed through GitHub [6] and Bioconductor [7]. The best way to use GSCA is to install and run it on users' own computers. To do so, users have to first download and install R from [8]. They also need to install at least one of the four PED compendia from Bioconductor [9, 10, 11, 12]. For instance, the compendium of Affymetrix Human Genome U133A Array can be installed by typing the following commands in R:

```
> source("http://bioconductor.org/biocLite.R")
> biocLite("Affyhgu133aExpr")
```

Next, users can install the latest GSCA from GitHub by typing the following commands in R:

```
> source("http://bioconductor.org/biocLite.R")
> biocLite("rhdf5")
> if (!require("devtools"))
+     install.packages("devtools")
> devtools::install_github("GSCA","zji90")
```

Alternatively, one can also install GSCA from Bioconductor following the instructions in [13]. However, since the Bioconductor only updates its packages twice per year, GSCA installed from Bioconductor may not be the most up-to-date version.

After installation, one can start the GUI by typing the following commands in R:

```
> library(GSCA)
> GSCAui()
```

Users are referred to a demonstration video at [14] to learn how to use GSCA. Users who are familiar with R programming can also run GSCA using command-line mode. The instructions for using GSCA as R commands are included in the GSCA package documentations.

For users who only want to do a one-time analysis, we also created an online web service to help them run GSCA directly online without the need to install R, GSCA or PED compendia on their own computers. The link to the web service is provided on the GSCA homepage at GitHub [6]. The online version, however, can be slow depending on the job load of the web server.

4 Defining POI Using Formulas

In GSCA, the POI can also be defined using formulas such as "Gene set A < ((Gene set B + Gene set C)/2)". The formulas can be defined using the arithmetic and logical operators in R following the grammar of R programming language. For example, one can use arithmetic operators such as $+$, $-$, $*$, $/$ and logical operators such as '<', '<=', '>', '>=', '&' (AND), '|' (OR), '!' (NOT), etc. To demonstrate, Supplementary Figure 2A shows an analysis of MYC and its 51 core target genes as an example. In this example, we used the following formula to define POI: $(MYC + 2)^2 + (MYC\_TG - 2)^2 * 10 < 4$. This POI defines an ellipse and is similar to the POI interactively specified in Figure 3E. Supplementary Figure 2A shows the samples selected by this formula-defined POI. The enriched biological contexts are shown in Supplementary Figure 2B,C. Once again, Wilms tumor was found in the enriched contexts, similar to the results from the interactive POI analysis in Figure 3E,G. Supplementary Figure 2D shows another example in which we analyzed two metabolic gene sets, glycolysis and fatty acid oxidation, used in Example II and Figure 5. Here, we defined the POI using formula "$Glycolysis < Fattyacid$ & $Fattyacid > 4$". The POI defined by this formula is very similar to the POI interactively defined in Figure 5B. Supplementary Figure 2D shows the selected samples, and Supplementary Figure 2E,F shows that these samples were enriched in liver, consistent with the interactive POI analysis in Figure 5B. When users define POI using formulas, they can also use R functions in the formula. This is demonstrated in Example IV and Supplementary Figure 11, where the POI is defined by "$MYC < quantile(MYC, 0.1)$ & $Mitochondria > quantile(Mitochondria, 0.9)$ & $Glycolysis > quantile(Glycolysis, 0.9)$ & $Fattyacid > quantile(Fattyacid, 0.9)$". Here quantile() is a function in R to determine quantiles.

5 Analysis of Sensitivity

Generally speaking, evaluating sensitivity for GSCA is not easy. Unlike FDR which can be estimated by performing a targeted literature search based on the GSCA reported biological contexts (which is tedious but still feasible), objectively evaluating sensitivity requires one to have a comprehensive or large enough list of true relationships between biological contexts and a POI which is seldom available. With this practical constraint, we used the MYC analysis (i.e., Example I) to obtain a rough idea of sensitivity because MYC is relatively well-studied in the literature. Even for this well-studied TF, its functional contexts with direct experimental proof has not been systematically cataloged before. For evaluation purpose, we instead obtained a list of 16 biological contexts reviewed by [15] that are MYC-related based on previously documented MYC overexpression or amplification. MYC TF is likely (but not guaranteed) to play a functional role in these contexts. These 16 contexts were compiled independent of our GSCA analysis. We used them as "gold standard" to study sensitivity by evaluating what proportion of them was discovered by GSCA in our MYC analysis using the default POI (i.e., high MYC and high MYC target gene set activity in Example I). It turns out that 14 of the 16 gold standard contexts were present in our PED compendium, and 2 contexts were not covered by our PED compendium (Supplementary Table 3). In the GSCA analysis, a total of 127 biological contexts appeared in the default POI region (i.e., these contexts had at least one sample showing the POI). These 127 contexts covered 13 gold standard contexts, representing 92.86% of the 14 gold standard contexts available in our PED compendium and 81.25% of all 16 gold standard contexts. Not all of the 127 contexts appearing in the POI region passed the default significance cutoff defined by adjusted p-value < 0.05 and fold change > 1.5. Among these 127 contexts, 30 contexts passed the cutoff and were reported by GSCA. These 30 contexts covered 7 gold standard contexts, translating into a sensitivity of 50% (7/14) for recovering the 14 gold standard contexts available in our PED compendium and a sensitivity of 43.75% (7/16) for recovering all 16 gold standard contexts. When interpreting these sensitivities, it is important to keep several things in mind.

First, besides the 7 significant contexts, 6 other gold standard contexts appeared in the POI region but they did not pass the default reporting cutoff. A careful examination shows that these contexts had large fold change but they did not pass Fisher's exact test because of small sample size. Here the sample size refers to the total number of samples a context has in the PED compendium. Supplementary Figure 3A shows the sample size distribution for five different types of biological contexts: (1) contexts with at least one sample in the POI region that passed the default significance cutoff and hence reported by GSCA ("Contexts with POI, Significant"), (2) contexts with at least one sample in the POI region that did not pass the default significance cutoff and hence not reported by GSCA ("Contexts with POI, Not Significant"), (3) the 7 gold standard MYC contexts with at least one sample in the POI region that were reported by GSCA ("Known MYC contexts with POI, Significant"), (4)

the 6 gold standard MYC contexts with at least one sample in the POI region that were not reported by GSCA ("Known MYC contexts with POI, Not Significant"), (5) all contexts in the PED compendium ("All contexts"). This figure shows that the 6 gold standard MYC contexts appearing in the POI region but not passing the significance cutoff had smaller sample size (median sample size = 3) compared to the significant contexts reported by GSCA. The small sample size has limited the statistical power for detecting these contexts because one cannot get sufficiently small p-values. These 6 contexts, however, had relatively large fold change (Supplementary Figure 3B, median = 11.2, min = 3.7, max = 13.4). As our PED compendium continues to grow, the sample size of each context is expected to grow. This can potentially increase the statistical power for detecting the contexts that are currently missed by GSCA. To provide a better picture, we performed the following simulation study based on the MYC example. For each biological context c in our Affymetrix Human Genome U133A compendium, we multiplied the total number of samples (n_c) as well as the number of samples with POI (k_c) by a factor α (α= 1.5 or 2) to mimic the sample size increase, and we rounded αn_c and αk_c to their closest integers. As sample size increases, one also expects the number of contexts in the PED compendium to increase. Therefore, we also simulated a number of new contexts and added them to the compendium so that the total number of contexts in the compendium became βC after adding the new contexts. Here C is the original number of contexts in the compendium, and $\beta = \alpha$ or 2α. This yields four (α, β) combinations (1.5, 1.5), (1.5, 3), (2, 2), (2, 4). To keep the sensitivity and FDR calculation conservative, the newly generated contexts were assumed to be unrelated to MYC (i.e., we assume that when PED expands, all newly added contexts are noise). Accordingly, in order to generate these new contexts, we first excluded all MYC-related contexts from our PED compendium. The excluded contexts include all the literature supported MYC contexts in Supplementary Table 3 and the 16 pre-compiled gold standard MYC contexts used for sensitivity calculation. After excluding these MYC-related contexts, we randomly sampled the remaining contexts with replacement and used their updated sample sizes and POI sample counts $(\alpha n_c, \alpha k_c)$ to serve as the sample sizes and POI sample counts for the new contexts. After adding the new contexts to the existing PED compendium, the total number of samples in the PED compendium was increased from N to approximately $\alpha\beta N$. For example, if the sample size of each context is increased from n_c to $1.5n_c$ and the number of contexts is increased from C to $1.5C$, then the total sample number will be increased to approximately $1.5 * 1.5N = 2.25N$ (i.e., approximately doubled). For each combination of α and β, the whole simulation process was repeated 5 times. For each simulation, we recalculated the sensitivity based on the 16 gold standard MYC contexts. The FDR was recomputed following the same procedure described previously based on the MYC-context relationships supported by the literature listed in Supplementary Table 3. Here we treated all simulated new contexts as false discoveries. Supplementary Figure 4 shows the sensitivity and FDR for each (α, β) combination. For all combinations of α and β, the sensitivity increased to 81.25% (i.e., all 13 gold standard contexts appearing in

the POI region became significant) and the FDR stayed around 30% to 35%. This simulation shows that with the continual growth of PED compendium, the sensitivity of GSCA can be greatly increased.

Second, although the data currently available in GSCA may not provide the power to discover everything through Fisher's tests, our examples in the article such as the MYC - Ewing tumor relationship demonstrate that one can still make many new predictions and discoveries using the current GSCA. These new findings can greatly expand people's current knowledge about genes and pathways. Without a tool like this, large amounts of valuable information in PED will remain unutilized. Compared to not using these data at all, being able to use them to make new discoveries already represents a significant progress even if one may only partially reconstruct the truth. Clearly, it is important to continually expand our data collection and increase the power of GSCA. However, data collection is a continual effort. It is not necessary to wait until enough data from all contexts are collected (which may take many years of time) before making this tool available to allow people benefit from it.

Third, Fisher's test is mainly used to screen for biological contexts whose association with POI cannot be trivially explained by chance. As shown above, biological contexts truly associated with the POI but without sufficient support from the PED (e.g., due to small sample size) may be filtered out by such tests. If users only want to use GSCA to explore the data (e.g., screen for "possible hypotheses" rather than "hypotheses that are unlikely to occur by chance"), or if they have other types of data to further screen or validate the biological contexts reported by GSCA, they may ignore the Fisher's test. For instance, in the MYC example, one may ask GSCA to return all contexts appearing in the POI region and then use other data sources to determine which biological contexts are most relevant. In this way, 13 (81.25%) of the 16 gold standard MYC contexts would pass the initial GSCA screen.

6 Impact of Noisy Gene Set

A question of interest is what will happen if one uses a gene set that is not well studied and contains some noise. In general, for a poorly studied gene set, it is difficult to compute the sensitivity and FDR since the true relevant biological contexts are largely unknown. Thus, in order to answer this question, we again took the advantage of the relatively well-studied MYC example. We replaced x percent ($x = 25\%, 50\%, 75\%, 90\%$) of genes in the MYC target gene set with genes randomly drawn from the microarray. We then reran GSCA (default POI) using MYC and the new target gene set. We computed sensitivity using the 16 pre-complied MYC contexts as described above in the "Analysis of sensitivity" section, and computed FDR based on the support from literature listed in Supplementary Table 3. For each x, Supplementary Figure 5 shows the number of biological contexts reported at the Bonferroni adjusted p-value cutoff of 0.05, and the corresponding sensitivity and FDR. The simulation was repeated 5 times for each x. The figure shows the mean and range of the five

runs. These results show that replacing $\leq 50\%$ of the MYC target genes by noise only resulted in small changes in the number of reported contexts, sensitivity and FDR. The performance decrease was obvious only when $> 75\%$ of MYC target genes were replaced by noise. When the noise percentage was bigger than 75%, increasing the noise level decreased the number of significant contexts reported by GSCA. At the same time, the sensitivity also decreased, while the empirical FDR increased. This analysis indicates that GSCA is relatively robust to noises in the gene sets.

7 Demonstration of Non-integer weights

While $+1$ and -1 are the most commonly used weights in GSCA, weights other than ± 1 can sometimes help users to obtain more accurate results. To demonstrate, we used GSCA to analyze a gene set derived from a study of sonic hedgehog (SHH) signaling pathway in mouse developing forelimbs [16]. SHH signaling is known to play an important role in the limb bud development. The SHH protein has a higher concentration in posterior portion of the forelimbs compared to their anterior portion. Previously, we have generated gene expression data for both anterior and posterior portions of mouse forelimbs using Affymetrix Mouse Exon 1.0ST Arrays. These data are stored in GEO (accession no.: GSE11063). Using these data, one can derive SHH target genes by searching for genes that have significantly higher expression in posterior forelimbs compared to anterior forelimbs. To demonstrate GSCA, we downloaded the GeneBASE [17] normalized gene expression profiles for this study from GEO. We then applied limma [18] to the normalized and log-transformed data to detect genes up-regulated in the posterior forelimbs. 18 up-regulated genes were obtained at the 10% FDR cutoff. The number of differentially expressed genes was small because this dataset has relatively low signal-to-noise ratio. The data contained high technical noise because developing limb buds are tiny and precisely cutting forelimbs into an anterior part and a posterior part according to a fixed ratio of area is difficult. The 18 genes found above are expected to contain SHH target genes. Treating these genes as one gene set, we performed GSCA analysis using two different weighting schemes.

1. Simple weighting: $+1$ were used as weights for all genes since they are all up-regulated in posterior forelimbs.
2. Complex weighting: weights were computed based on the 18 moderated t-statistics reported by limma. The moderated t-statistics were linearly scaled to the [0,1] interval such that after scaling, the minimum and maximum values of the 18 moderated t-statistics became 0 and 1 respectively. These scaled statistics were then used as weights.

For both weighting schemes, GSCA was run in the one gene set mode using the default POI (i.e., gene set activity $\geq$ mean + 1*SD). The results are shown

in Supplementary Figure 6 and Supplementary Table 4. While these two different weighting schemes reported a number of common biological contexts, there were also clear differences. In particular, medulloblastoma, a known context of SHH function [19, 20], was identified by the complex weighting scheme as the top significant context (Supplementary Table 4, the context ranked no.1 in the complex weighting sheet), whereas the simple weighting scheme did not find it. The complex weighting scheme also discovered cerebellar tumor from Olig2-tva-cre:SmoM2 mice (rank no.10) which was not reported by the simple weighting approach. The SMO protein is a core component in the SHH pathway that relays the SHH signal. SmoM2 is a gain-of-function mutant of *Smo* gene that turns on the SHH pathway constitutively [16]. Therefore, SHH target genes are expected to be turned on in this context. SHH is also known to play important roles in embryonic head development [21]. While the complex weighting scheme found embryonic head tissues to be significant, they were not reported by the simple weighting scheme. The contexts found by the simple weighting scheme but not by the complex weighting scheme include adult liver, tail epidermis and cervix. Currently the SHH signaling is not known to have significant functions in these contexts. This example illustrates that a complex weighting scheme can be useful in certain applications by producing more accurate results.

8 GSCA Analysis of *Gli1* and *Nanog*

Like *Oct4*, *Nanog* is a critical pluripotency marker that is expressed in embryonic stem cells [22]. Neural stem cells also express *Nanog*, and require secreted Hedgehog signaling to maintain proliferation and to regulate differentiation [23]. While the mechanisms of this regulation are likely to be complex, two recent studies have shown that Hedgehog signaling helps regulate NANOG by activating GLI transcription factors [24, 25]. Given the widespread expression of *Nanog* in pluripotent cell populations, we asked if *Nanog* and the Hedgehog target gene *Gli1* might be co-expressed in other biological contexts using GSCA. Samples with high *Gli1* and low *Nanog* expression were enriched in medulloblastoma (Supplementary Figure 8C), consistent with the known role of Hedgehog signaling in a major subset of this tumor type [20]. Samples with high *Gli1* and high *Nanog* expression were enriched in various types of stem cells, including embryonic stem cells where they had not been previously associated (Supplementary Figure 8A). Intriguingly, there is also a group of samples with high *Gli1* and medium level of *Nanog* expression. These samples, which cannot be analyzed using ChIP-PED, were enriched in fetal mouse testes (Figure 4B, Supplementary Figure 8B). This points to a new biological context to potentially study GLI1 and NANOG interactions.

9 GSCA Analysis of *Oct4*

Supplementary Figure 9 shows an analysis of *Oct4* in the compendium of Affymetrix Mouse Genome 430 2.0 Array. Here the POI is defined interactively based on setting sliders on a plot that sorts the samples according to the gene set activity (Supplementary Figure 9A). One may add multiple sliders to define multiple intervals and then use their union as the POI. Samples whose gene set activity falls within these intervals will be selected for enrichment analysis. The samples chosen in Supplementary Figure 9A had either high or medium *Oct4* expressions. The enriched biological contexts mainly included undifferentiated or differentiating embryonic stem cells (Supplementary Figure 9B), consistent with the master regulator role of *Oct4* in stem cells.

10 Caveats in Statistical Inference

10.1 Interpretation of statistical significance in interactive analysis

In GSCA, the interactive POI function is mainly provided to help users conveniently explore the data and generate hypotheses. Users have to be careful when interpreting the adjusted p-values from the interactive analysis. The interpretation depends on how the analysis is carried out.

In one common scenario (scenario 1), a group of samples shows an interesting expression pattern (e.g., the POI in Figure 5B) but the pattern cannot be easily defined using a few simple cutoffs like "gene set activity > 1.0". In such a scenario, defining POI interactively (e.g., by drawing a polygon) allows one to conveniently select those samples. Here, one has a rough idea of what expression pattern is of interest based on looking at the histogram, scatter plot or heat map. One also has a specific question in mind, that is, "what biological contexts are associated with these samples that show this interesting pattern". The interactive POI is primarily used to help one formalize the question, and the POI is defined before one looks at the GSCA results (i.e., before knowing what biological contexts are enriched). For applications of this type, the adjusted p-value reported by GSCA can be used as a statistical significance measure as long as one does not repeatedly tune the POI based on the GSCA results to make the findings "more significant".

In another scenario (scenario 2), one does not have a clear idea of what expression patterns are interesting, and one wants to repeatedly try different POIs until something "significant" is found. For example, one may tune the POI after looking at GSCA results to make the reported p-values smaller. In such a scenario, the adjusted p-values reported by GSCA no longer reflect the true error rates because of data snooping, and therefore they can no longer be interpreted as a formal statistical significance measure. Large adjusted p-values may still be used by users to filter out biological contexts that lack sufficient data support for their association with the POI. However, small adjusted p-values do

not necessarily imply that the discoveries are statistically significant, and users should use other independent sources of information to help with determining whether they are real signals or just noise.

Measuring statistical significance of a data snooping procedure that involves human-machine interactions is still an open problem in statistics. Moreover, in practice when users perform the analyses, the GSCA software does not know which scenario users are in. Therefore, the software will always report the adjusted p-values which will be useful for users in scenario 1. However, these p-values should not be interpreted as a formal statistical significance measure in scenario 2. It is important that users are aware of the differences between these two scenarios and their implications in order to avoid misusing or misinterpreting the adjusted p-values.

10.2 Correlations among samples

GSCA currently uses Fisher's exact test to filter out biological contexts for which the available data are not enough to support that their occurrence in the POI region is nonrandom. Users should bear in mind that p-values from hypothesis testing are always based on assumptions made in the null hypotheses, and p-values should be interpreted with respect to these assumptions. Fisher's exact test assumes that samples are independent. Therefore, the adjusted p-values reported by GSCA characterize statistical significance under this assumption. Since samples may not be perfectly independent in real data, we always recommend users to use other independent sources of information to further validate biological contexts with small adjusted p-values whenever possible. At the same time, users may still use large adjusted p-values to help them filter out contexts without strong data support.

We also explored the possibility to evaluate statistical significance without assuming sample independence. Statistical significance can be evaluated using permutations. There are two basic ways to do permutations: permuting samples or permuting genes. Permuting samples retains the correlation structure among genes, but it can break the correlation among samples. Permuting genes retains the correlation structure among samples, but it can break the correlation among genes. Conceptually, Fisher's test is based on permuting samples' biological context labels. Permuting data in this way does not change the gene expression landscape, that is, histograms such as Figure 6A, scatter plots such as Figure 3D and E, or heat maps such as Figure 7A will remain unchanged after the permutation. However, the permutation changes how the samples are labeled and hence the distribution of each biological context in the expression landscape. This is the approach currently used by GSCA.

Instead of permuting samples, we tried to permute genes in order to keep the inter-sample correlation. In this approach, genes' labels are permuted, but we keep samples' biological context labels unchanged. The approach retains the correlation structure among samples, but it breaks the correlation among genes. Permuting genes is equivalent to replacing the test gene sets by random gene sets. In other words, consider a POI defined using one or multiple gene

sets. Suppose there are n samples in the PED compendium annotated with biological context c, and among them k samples have the POI. In order to test whether these k samples represent a significant enrichment of context c in the POI, we replace each original gene set with a random gene set having the same number of randomly selected genes. This is done by permuting all gene labels in our microarray compendium. After permutation, the gene set activities are recalculated. Suppose a total of J permutations are carried out, and k_j represents the number of samples from the biological context c whose gene set activities in permutation j show the original POI. The raw p-value for testing the association between context c and the POI is then calculated as $\frac{\sum_j \delta(k_j \geq k)}{J}$, where $\delta(.)$ is an indicator function. When testing this approach, we found that it has a problem, that is, the gene expression landscape of the random gene sets usually is very different from the gene expression landscape of the original gene sets. As a result, the original POI used to analyze the test gene sets is no longer meaningful after permutation. Supplementary Figure 12 provides an example of permuting genes for glycolysis and fatty acid gene sets in Example II, Figure 5B. Supplementary Figure 12A shows the original gene set activities. The POI was defined by a polygon interactively drawn to select a group of samples with high fatty acid pathway activity but medium level of glycolysis activity. One is interested in these samples since they are separated from the main cloud of other samples. This POI region contained samples from 17 different contexts. Supplementary Figure 12B-D shows gene set activities obtained from three different gene permutations. In all three cases, the gene set activity landscape was substantially different from the original landscape, and the original POI region did no longer contain any sample. In fact, when we repeated the gene permutation procedure, this phenomenon occurred in all permutations. As a result, the Bonferroni corrected p-values based on permuting genes were 0 for all 17 contexts appearing in the original POI region, including contexts with only one or two samples in the PED compendium. By contrast, in Fisher's test, only 9 of the 17 contexts passed the adjusted p-value < 0.05 cutoff. Thus, permuting genes has led to much smaller p-values and more optimistic conclusions than permuting samples. This was simply because permuting genes changed the expression landscape and made the original POI irrelevant in the new landscape. This phenomenon is very common and we observed it for almost all other gene sets we have analyzed. Thus, even though permuting genes may allow one to retain the inter-sample correlation, it is empirically less stringent than Fisher's exact test, likely because the gene independence assumption is more unrealistic than the sample independence assumption. For this reason, we adopted the more stringent Fisher's exact test in GSCA.

An alternative way to handle correlation among samples is to directly model the correlation and incorporate the samples' variance-covariance structure into the analysis. For example, one may borrow the idea used by ROAST [26] where the sample variance-covariance is accounted for through a sample weight matrix, or the idea of CAMERA [27] that directly estimates the variance inflation caused by correlation. In order to use these approaches, however, one needs

to know how samples are correlated. More precisely, if one uses a samplewise statistic z_i to indicate whether sample i shows the POI ($z_i = 1$) or not ($z_i = 0$), then one needs to know the variance-covariance structure of z_i among different samples. There are a number of challenges here. First, z_i is a function of genes' expression values (denoted as $\mathbf{y}_i$) of sample i. The function is determined by the POI. This function is usually non-linear and can be very complex since POI can be arbitrary. The correlation structure of z_i is not equivalent to the correlation structure of $\mathbf{y}_i$. In other words, the correlation between two samplewise statistics z_i and z_j from two samples i and j cannot be simply estimated using the correlation between the two gene expression vectors $\mathbf{y}_i$ and $\mathbf{y}_j$. These two correlations can be very different, and there is no known method with theoretical guarantee that can convert the correlation between $\mathbf{y}_i$s to correlation between z_is. Moreover, since each sample only has one observed value of z_i for a given PED compendium and a given POI, directly estimating the correlation among z_is is difficult due to the lack of degree of freedom. Second, the variance-covariance matrix of the PED samples is a high-dimensional matrix due to the size of the PED compendium. Even if one can use the correlation structure of $\mathbf{y}_i$s to infer the correlation structure of z_is, estimating such a high-dimensional variance-covariance matrix is known to be challenging in statistics. Third, for exploratory analysis, it is important that the computation is fast and does not take hours to run. Unfortunately, efficiently estimating and operating on a high-dimensional variance-covariance matrix is difficult, and currently we have not yet found a statistically and computationally efficient solution that is suitable for the application settings of GSCA. For these reasons, methods such as ROAST and CAMERA currently cannot be directly adapted to GSCA. Whether one can develop similar methods suitable for GSCA that can appropriately and efficiently handle both the inter-sample correlation and the inter-gene correlation is an important topic for future investigation.

References

[1] McCall, M.N., Jaffee, H.A., Zelisko, S.J., Sinha, N., Hooiveld, G., Irizarry, R.A., and Zilliox, M. J. (2014) The Gene Expression Barcode 3.0: improved data processing and mining tools. *Nucleic Acids Res.*, **42(D1)**, D938–D943.

[2] Wu, G., Yustein, J.T., McCall, M.N., Zilliox, M., Irizarry, R.A., Zeller, K., Dang, C.V. and Ji, H. (2013) ChIP-PED enhances the analysis of ChIP-seq and ChIP-chip data. *Bioinformatics.*, **29(9)**, 1182-1189.

[3] Liu, X., Yu, X., Zack, D. J., Zhu, H., and Qian, J. (2008) TiGER: a database for tissue-specific gene expression and regulation. *BMC Bioinformatics.*, **9(1)**, 271.

[4] List of common cell types from ENCODE.

http://genome.ucsc.edu/encode/cellTypes.html

[5] Walz, S., Lorenzin, F., Morton, J. Wiese, K. E., von E. B., Herold, S., Rycak, L., Dumay-Odelot, H., Karim, S., Bartkuhn, M. and et al. (2014) Activation and repression by oncogenic MYC shape tumour-specific gene expression profiles. *Nature.*, **511(7510)**, 483–487.

[6] GSCA home page on Github.
https://github.com/zji90/GSCA

[7] Bioconductor.
http://www.bioconductor.org/install/

[8] R-project.
http://www.r-project.org/

[9] Affyhgu133aExpr Bioconductor page.
http://www.bioconductor.org/packages/release/data/experiment/html/Affyhgu133aExpr.html

[10] Affyhgu133Plus2Expr Bioconductor page.
http://www.bioconductor.org/packages/release/data/experiment/html/Affyhgu133Plus2Expr.html

[11] Affyhgu133A2Expr Bioconductor page.
http://www.bioconductor.org/packages/release/data/experiment/html/Affyhgu133A2Expr.html

[12] Affymoe4302Expr Bioconductor page.
http://www.bioconductor.org/packages/release/data/experiment/html/Affymoe4302Expr.html

[13] GSCA Bioconductor page.
http://www.bioconductor.org/packages/release/bioc/html/GSCA.html

[14] GSCA Demo Video.
https://www.youtube.com/watch?v=wqv_dmlxdcI

[15] Vita, M. and Henriksson, M. (2006) The Myc oncoprotein as a therapeutic target for human cancer. *Semin Cancer Biol.*, **16(4)**, 318–330.

[16] Vokes, S.A., Ji, H., Wong, W.H. and McMahon, A.P. (2008) A genome-scale analysis of the cis-regulatory circuitry underlying sonic hedgehog-mediated patterning of the mammalian limb. *Genes Dev.*, **22(19)**, 2651-2663.

[17] Kapur, K., Xing, Y., Ouyang, Z. and Wong, W.H. (2007) Exon arrays provide accurate assessments of gene expression. *Genome Biol.*, **8(5)**, R82.

[18] Smyth, G.K. (2005) Limma: linear models for microarray data. *Bioinformatics and computational biology solutions using R and Bioconductor*, **397-420**, Springer New York.

[19] Lee, E.Y., Ji, H., Ouyang, Z., Zhou, B., Ma, W., Vokes, S.A., McMahon, A.P., Wong, W.H. and Scott, M.P. (2010). Hedgehog pathway-regulated gene networks in cerebellum development and tumorigenesis. *Proc. Natl. Acad. Sci. USA*, **107(21)**, 9736–9741.

[20] Northcott, P.A., Korshunov, A., Pfister, S.M. and Taylor, M.D. (2012) The clinical implications of medulloblastoma subgroups. *Nat Rev Neurol.*, **8(6)**, 340–351.

[21] Allen, B.L., Tenzen T. and McMahon A.P. (2007) The Hedgehog-binding proteins Gas1 and Cdo cooperate to positively regulate Shh signaling during mouse development. *Genes Dev.*, **21(10)**, 1244-57.

[22] Martello, G., Bertone, P. and Smith, A. (2013) Identification of the missing pluripotency mediator downstream of leukaemia inhibitory factor. *EMBO J.*, **32(19)**, 2561–2574.

[23] Álvarez-Buylla, A. and Ihrie, R.A. (2014) Sonic Hedgehog Signaling in the Postnatal Brain. *Semin Cell Dev Biol.*

[24] Po, A., Ferretti, E., Miele, E., De Smaele, E., Paganelli, A., Canettieri, G., Coni, S., Di Marcotullio, L., Biffoni, M., Massimi, L. and et al. (2010) Hedgehog controls neural stem cells through p53-independent regulation of Nanog. *EMBO J.*, **29(15)**, 2646–2658.

[25] Zbinden, M., Duquet, A., Lorente-Trigos, A., Ngwabyt, S.-N., Borges, I. and Ruiz i Altaba, A. (2010) NANOG regulates glioma stem cells and is essential in vivo acting in a cross-functional network with GLI1 and p53. *EMBO J.*, **29(15)**, 2659–2674.

[26] Wu, D., Lim, E., Vaillant, F., Asselin-Labat, M., Visvader, J. E. and Smyth, G. K. (2010) ROAST: rotation gene set tests for complex microarray experiments. *Bioinformatics.*, **26(17)**, 2176–2182.

[27] Wu, D. and Smyth, G. K. (2012) Camera: a competitive gene set test accounting for inter-gene correlation. *Nucleic Acids Res.*, **40(17)**, e133–e133.

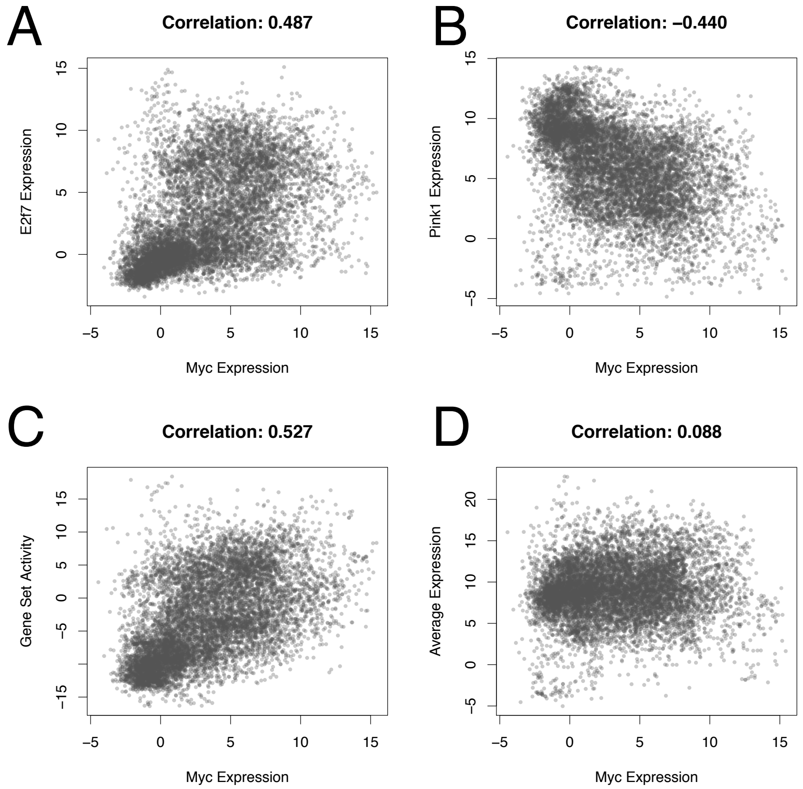

Supplementary Figure 1: Illustration of using positive and negative weights in computing gene set activity. A: The expression of *E2f7* (y-axis) is positively correlated with *Myc* expression (x-axis). The Pearson correlation coefficient is shown above the plot. B: *Pink1* is negatively correlated with *Myc*. C: When the weight for *E2f7* is 1 and the weight for *Pink1* is -1, the gene set activity defined using the weighted average of *E2f7* and *Pink1* expression is positively correlated with *Myc*, and the correlation is stronger than (A) and (B). D: When the weights for *E2f7* and *Pink1* are both equal to 1, the gene set activity is equal to the average expression of *E2f7* and *Pink1*. It is not strongly correlated with *Myc*.

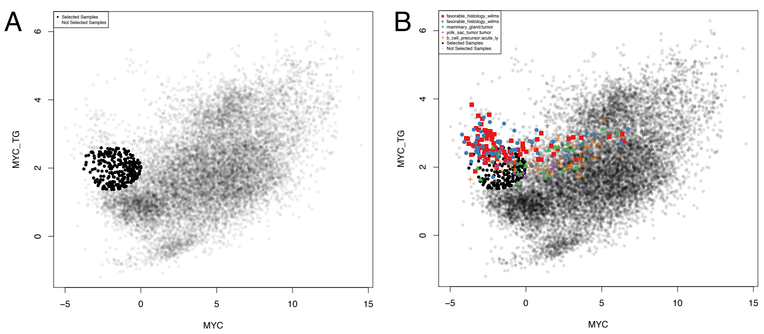

C

Rank	Active	Total	FoldChange	Adj.Pvalue	SampleType	ExperimentID
1	33	90	13.823	6.31*E-27	favorable_histology_wilms_tumor:nonrelaps	GSE10320
2	18	53	12.715	3.7*E-13	favorable_histology_wilms_tumor:relapse	GSE10320
3	10	24	15.275	1.26*E-07	mammary_gland:tumor	GSE6596
4	9	18	18.094	1.38*E-07	yolk_sac_tumor:tumor	GSE10615
5	9	44	7.64	0.00111	b_cell_precursor:acute_lymphoblastic_leuke	GSE13280
6	60	1266	1.804	0.00164	breast:tumor	GSE12093;GSE1456;GSE
7	3	3	28.816	0.0122	hematopoeitic_stem_cell_cb_+:normal	GSE3823
8	3	3	28.816	0.0122	hematopoeitic_stem_cell_cb_++:normal	GSE3823
9	4	9	15.335	0.0359	cd14+_cells:normal	GSE11943

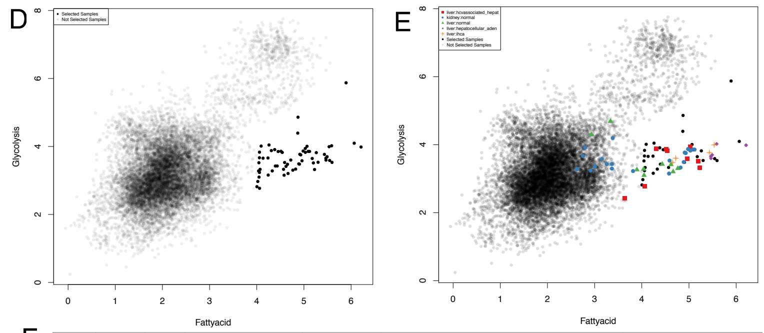

F

Rank	Active	Total	FoldChange	Adj.Pvalue	SampleType	ExperimentID
1	8	9	129.212	8.09*E-15	liver:hcvassociated_hepatocellular_carcinon	GSE14323
2	10	21	73.404	8.53*E-15	kidney:normal	GSE781;GSE6344
3	6	9	96.934	2.46*E-09	liver:normal	GSE2004;GSE2361
4	5	5	134.658	5.23*E-09	liver:hepatocellular_adenoma	GSE7473
5	4	4	129.312	9.05*E-07	liver:ihca	GSE11819
6	4	4	129.312	9.05*E-07	liver:non_tumoral	GSE7473
7	4	4	129.312	9.05*E-07	liver:tumor	GSE9536
8	4	6	92.366	1.35*E-05	small_intestine:normal	GSE13083
9	3	3	121.292	0.000154	lncap_cells:rti6413018,_24hr	GSE4636

Supplementary Figure 2: Illustration of defining POI using formulas. A: Analysis of MYC and its 51 core target genes in Example I. The POI is defined using formula "$(MYC+2)^2+(MYC\_TG-2)^2*10<4$". Samples in the POI region are highlighted by dark black. B: Top enriched biological contexts in (A) and all their samples are shown in color. C: The top two enriched biological contexts in (A) are both related to Wilms tumor. D: Analysis of glycolysis and fatty acid oxidation gene sets in Example II. The POI is defined using formula "Glycolysis $<$ Fattyacid & Fattyacid $>$ 4". Samples in the POI region are highlighted by dark black. E: Top enriched biological contexts in (D) and all their samples are shown in color. F: The top enriched biological contexts in (D) are liver.

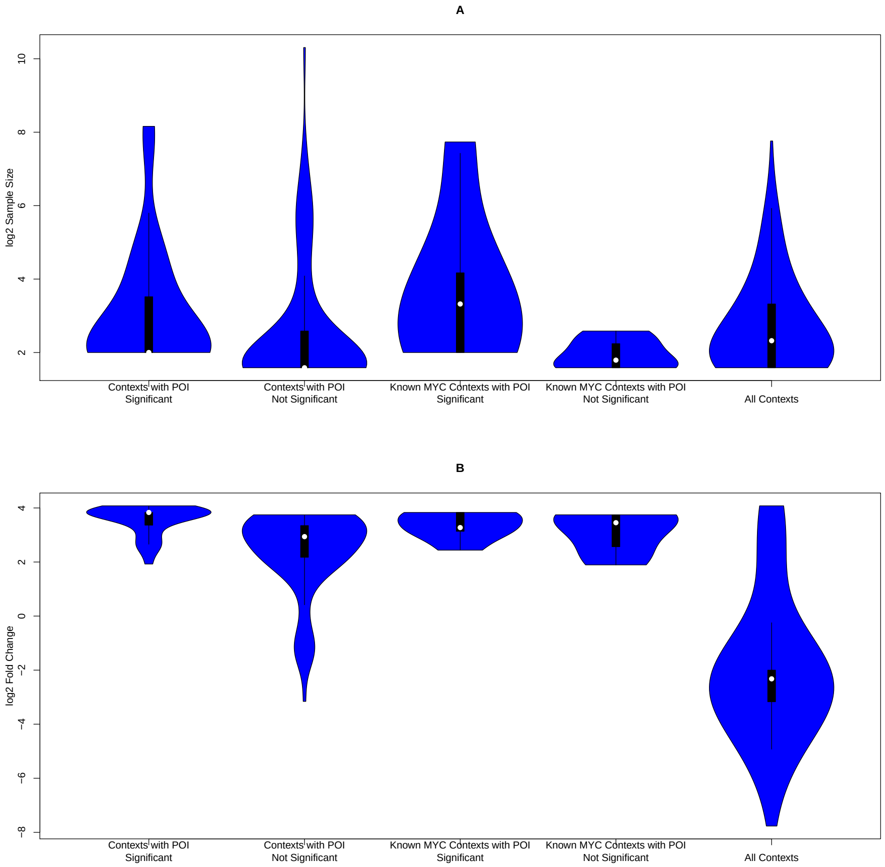

Supplementary Figure 3: Sample size and fold change distribution of five different types of biological contexts. A: Violin plots showing the distribution of $\log_2(sample\ size)$ (i.e., $\log_2(n_c)$). B: Violin plots showing the distribution of $\log_2(fold\ change)$ (i.e., $\log_2(f_c)$). From left to right, the five biological context types are (1) contexts appearing in the POI region that passed the significance cutoff of GSCA, (2) contexts appearing in the POI region that did not pass the GSCA significance cutoff, (3) the 7 gold standard MYC contexts appearing in the POI region that passed the GSCA significance cutoff, (4) the 6 gold standard MYC contexts appearing in the POI region that did not pass the GSCA significance cutoff, (5) all contexts in the PED compendium.

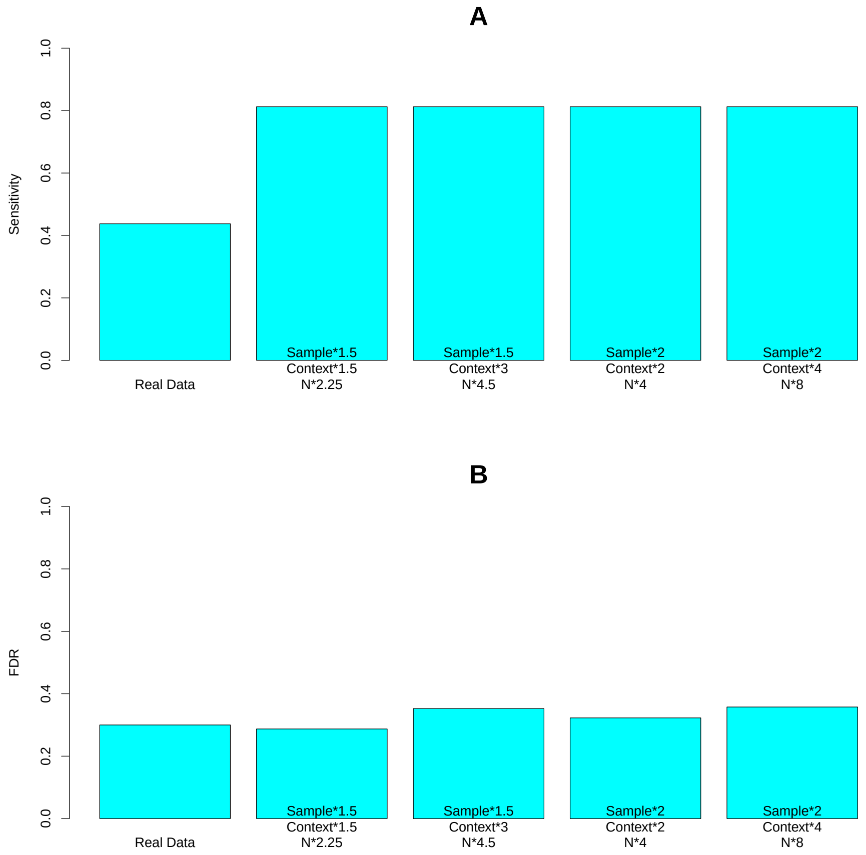

Supplementary Figure 4: Sensitivity and FDR of GSCA in simulations with increasing sample size. In the simulation study, the sample size per context is increased by multiplying a factor α, and the number of context is increased by multiplying another factor β. The total sample count in the compendium is approximately $\alpha\beta N$ after simulation where N is the original total sample count. For each (α, β), the simulation was run five times, and the average performance of these five runs is shown. A: Sensitivities for detecting the 16 gold standard MYC contexts in the original MYC analysis (Example I, default POI) and four simulations with different (α, β). B: FDR.

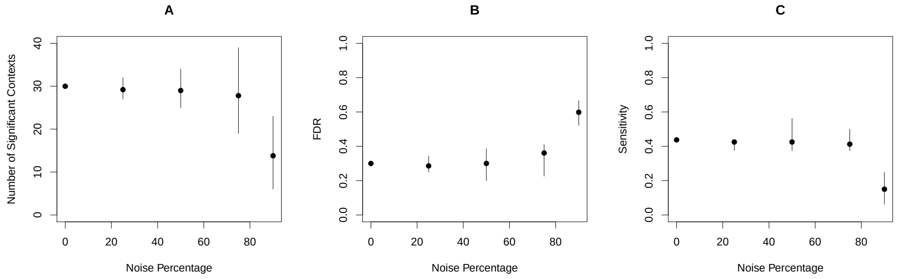

Supplementary Figure 5: GSCA performance in the MYC analysis (Example I, default POI) after replacing certain percentage (0%, 25%, 50%, 75%, 90%) of the 51 MYC target genes with noise (i.e., randomly chosen genes). A: The number of significant contexts reported by GSCA at its default cutoff. B: FDR. C: Sensitivity for detecting the 16 gold standard MYC contexts. For each noise percentage, the noise replacement process was repeated 5 times independently. The plots show the mean and range of the five runs.

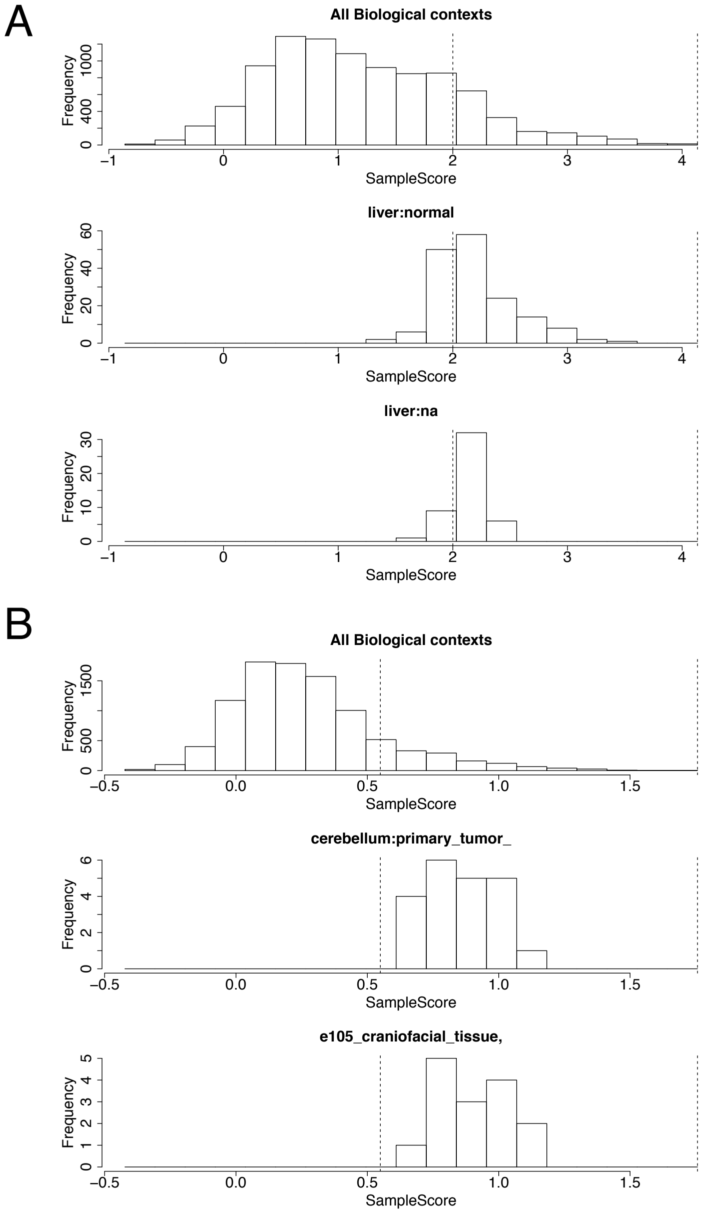

Supplementary Figure 6: GSCA analysis of the SHH gene set with the default POI using two different weighting schemes. A: Simple weighting scheme where all genes have equal weights. B: Complex weighting scheme where genes' weights depend on their moderated t-statistics.

A

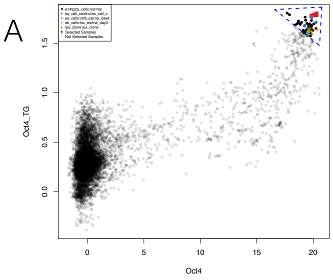

Rank	Active k_c	Total n_c	FoldChange $(Nk_c+K)/(Kn_c+K)$	Adj.Pvalue	SampleType	ExperimentID
1	8	8	118.259	7.1*E-15	e14tg2a_cells:normal	GSE4308;GSE4309
2	4	4	106.533	3.6*E-06	es_cell_control:es_cell_control	GSE14012
3	4	4	106.533	3.6*E-06	es_cells:ctr9_esirna_day4	GSE12078
4	4	4	106.533	3.6*E-06	es_cells:luc_esirna_day4	GSE12078
5	4	4	106.533	3.6*E-06	ips_clone:ips_clone	GSE14012
6	5	12	51.199	1.89*E-05	embryonic_stem_cells:r1,_undifferentia	GSE9563
7	5	13	47.542	3.06*E-05	embryonic_stem_cells:normal	GSE10476;GSE10573
8	3	3	99.938	0.00051	embryonic_stem_cell:es_cells_heterozy	GSE7141
9	3	3	99.938	0.00051	induced_pluripotent_cells:ips_cells_2_f	GSE10806
10	3	3	99.938	0.00051	v65_embryonic_stem_cells:normal	GSE8024

B

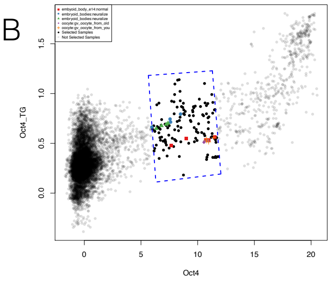

Rank	Active k_c	Total n_c	FoldChange $(Nk_c+K)/(Kn_c+K)$	Adj.Pvalue	SampleType	ExperimentID
1	5	5	61.988	5.05*E-07	embyoid_body_e14:normal	GSE8128
2	4	4	59.549	3.93*E-05	embryoid_bodies:neuralized_embryoid	GSE4936
3	4	4	59.549	3.93*E-05	embryoid_bodies:neuralized_embryoid	GSE4936
4	4	4	59.549	3.93*E-05	oocyte:gv_oocyte_from_old_mice_(66	GSE11667
5	4	4	59.549	3.93*E-05	oocyte:gv_oocyte_from_young_mice_(	GSE11667
6	4	4	59.549	3.93*E-05	oocyte:mii_oocyte_from_old_mice_(66	GSE11667
7	4	4	59.549	3.93*E-05	oocyte:mii_oocyte_from_young_mice_	GSE11667
8	4	8	33.083	0.00264	embryo:b6d2_2cell_scnt_embryos	GSE6595
9	4	8	33.083	0.00264	mef:neo(r)_mefs_derived_from_chime	GSE10871
10	3	3	55.89	0.00302	embryonic_stem_cells:cag_150_embry	GSE9760

Supplementary Figure 7: GSCA analysis of *Oct4* and its target genes. A: In the POI selected samples, *Oct4* and its target gene activities are both high. B: *Oct4* and its target gene activities are both at medium level. Plots on the left show the POIs. Tables on the right show the enriched biological contexts.

A

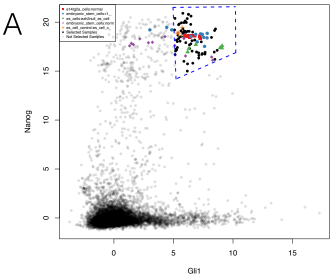

Rank	Active k_c	Total n_c	FoldChange $(Nk_c+K)/(Kn_c+K)$	Adj.Pvalue	SampleType	ExperimentID
1	8	8	81.127	1.78*E-13	e14tg2a_cells:normal	GSE4308;GSE4
2	9	12	63.176	3.71*E-13	embryonic_stem_cells:r1,_undifferent	GSE9563
3	6	6	78.265	1.79*E-09	es_cells:ezh2null_es_cells_at_day_0_	GSE12982
4	6	13	39.133	2.89*E-06	embryonic_stem_cells:normal	GSE10476;GSE
5	4	4	73.114	1.7*E-05	es_cell_control:es_cell_control	GSE14012
6	4	4	73.114	1.7*E-05	es_cells:ctr9_esirna_day4	GSE12078
7	4	4	73.114	1.7*E-05	es_cells:luc_esirna_day4	GSE12078
8	3	3	68.607	0.00161	embryonic_stem_cell:es_cells_heteroz	GSE7141
9	3	3	68.607	0.00161	embryonic_stem_cells:embryonic_ster	GSE8503
10	3	3	68.607	0.00161	embryonic_stem_cells:embryonic_ster	GSE8503

B

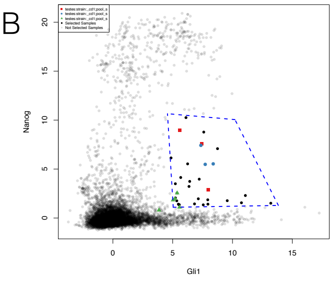

Rank	Active k_c	Total n_c	FoldChange $(Nk_c+K)/(Kn_c+K)$	Adj.Pvalue	SampleType	ExperimentID
1	3	3	239.5	3.36*E-05	testes:strain:_cd1;pool_size:_5;sex:_m;developmental_stage:_gd_12;theiler_stage20;somite_count:_na;developmental_landmark:_na	GSE4818
2	3	3	239.5	3.36*E-05	testes:strain:_cd1;pool_size:_na;sex:_m;developmental_stage:_gd_14;theiler_stage:22;somite_count:_na;developmental_landmark:_na	GSE4818
3	3	5	159.667	0.000335	testes:strain:_cd1;pool_size:_2;sex:_m;developmental_stage:_gd_11;theiler_stage:18;somite_count:_na;developmental_landmark:_na	GSE4818

C

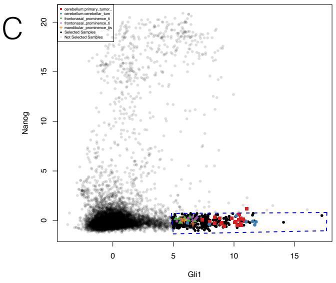

Rank	Active k_c	Total n_c	FoldChange $(Nk_c+K)/(Kn_c+K)$	Adj.Pvalue	SampleType	ExperimentID
1	20	21	23.719	5.85*E-25	cerebellum:primary_tumor_cells_isolated_from_conventional_patched_medulloblastomas	GSE12430
2	7	7	22.911	1.45*E-07	cerebellum:cerebellar_tumor_from_oli	GSE11859
3	7	7	22.911	1.45*E-07	frontonasal_prominence_tissue_e120:	GSE7759
4	7	7	22.911	1.45*E-07	frontonasal_prominence_tissue_e125:	GSE7759
5	7	7	22.911	1.45*E-07	mandibular_prominence_tissue_e100:	GSE7759
6	7	7	22.911	1.45*E-07	mandibular_prominence_tissue_e110:	GSE7759
7	7	7	22.911	1.45*E-07	mandibular_prominence_tissue_e120:	GSE7759
8	7	7	22.911	1.45*E-07	mandibular_prominence_tissue_e125:	GSE7759
9	7	7	22.911	1.45*E-07	maxillary_prominence_tissue_e115:nc	GSE7759
10	7	7	22.911	1.45*E-07	maxillary_prominence_tissue_e120:nc	GSE7759

Supplementary Figure 8: GSCA analysis of *Gli1* and *Nanog*. A: *Gli1* and *Nanog* are both highly expressed. B: *Gli1* is highly expressed and *Nanog* is expressed at medium level. C: *Gli1* is highly expressed and *Nanog* is lowly expressed.

A

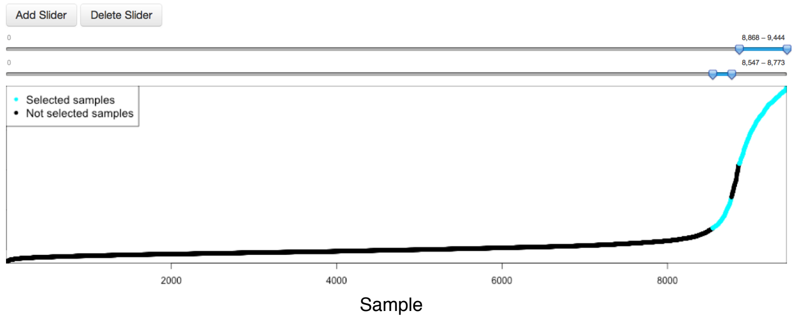

B

Rank	Active k_c	Total n_c	FoldChange $(Nk_c+K)/(Kn_c+K)$	Adj.Pvalue	SampleType	ExperimentID
1	20	20	11.666	1.71*E-19	inner_cell_mass_cell:gene_expressioı single_icm_cells_(e35)	GSE4307;GSE43
2	19	20	11.085	3.99*E-17	single_cell_from_blimpko_blimp1nullt	GSE11128
3	17	17	11.577	3.42*E-16	single_cell_from_lineagerestricted_pg	GSE11128
4	13	13	11.399	8.39*E-12	embryonic_stem_cells:normal	GSE9954;GSE10
5	12	12	11.337	1.05*E-10	embryoid_bodies:r1,_differentiated_c	GSE9563
6	12	12	11.337	1.05*E-10	embryonic_stem_cells:r1,_undifferen	GSE9563
7	15	23	7.666	1.41*E-08	testis:normal	GSE10744;GSE1
8	10	10	11.181	1.61*E-08	single_cell_from_posterior_mesoderm	GSE11128
9	8	8	10.955	2.48*E-06	e14tg2a_cells:10_pg_amplified	GSE4308;GSE43
10	8	8	10.955	2.48*E-06	e14tg2a_cells:normal	GSE4308;GSE43

Supplementary Figure 9: GSCA for one gene set illustrated using *Oct4*. A: Complex POI can be defined as the union of multiple intervals, specified interactively using multiple slider bars. B: The top 10 enriched biological contexts associated with the chosen POI.

A

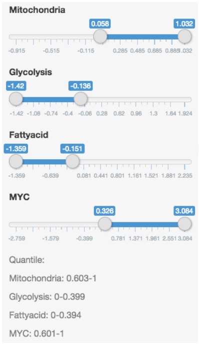

Rank	Active k_c	Total n_c	FoldChange $(Nk_c+K)/(Kn_c+K)$	Adj.Pvalue	SampleType	ExperimentID
1	5	15	270.574	1.44*E-09	293_hek_cells:6070%_conf	GSE1455

B

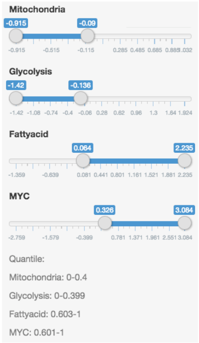

Rank	Active k_c	Total n_c	FoldChange $(Nk_c+K)/(Kn_c+K)$	Adj.Pvalue	SampleType	ExperimentID
1	36	286	12.576	4.92*E-28	blasts_and_mononuclear_ce	GSE1159
2	5	14	33.477	0.000113	whole_blood:grp	GSE2888
3	5	19	25.108	0.00063	liposarcoma_culture:gene_e	GSE12972
4	4	19	20.096	0.0219	liposarcoma_culture:gene_e	GSE12972

Supplementary Figure 10: GSCA for multiple gene sets. A: High MYC, high mitochondria, low fatty acid, and low glycolysis. B: High MYC, low mitochondria, high fatty acid, and low glycolysis.

A

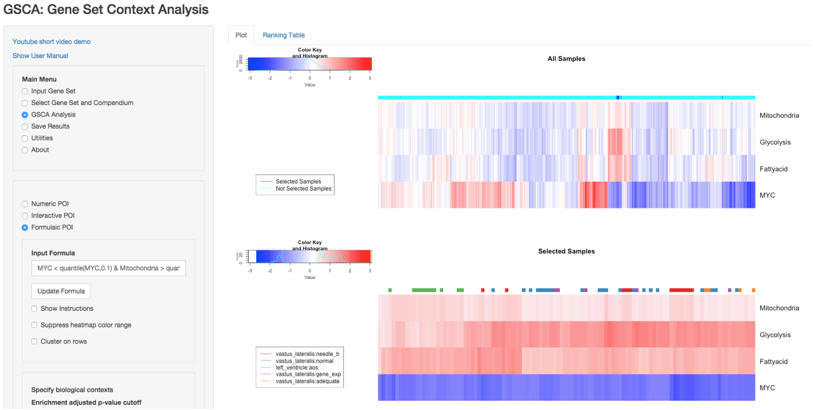

B

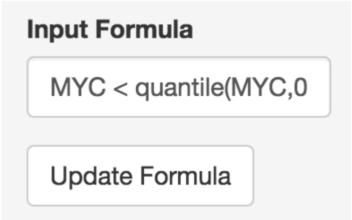

C

Rank	Active	Total	FoldChange	Adj.Pvalue	SampleType	ExperimentID
1	12	12	79.982	2.08*E-21	vastus_lateralis:needle_biops	GSE1786
2	21	98	18.372	1.19*E-18	vastus_lateralis:normal	GSE1786;GSE910
3	11	20	45.39	3.08*E-14	left_ventricle:aos	GSE10161
4	5	12	33.371	9.5*E-05	vastus_lateralis:gene_expres	GSE9105
5	4	6	49.608	0.000169	vastus_lateralis:adequate_pr	GSE8441
6	4	7	43.407	0.000391	left_ventricle:control	GSE10161
7	5	19	21.691	0.00131	vastus_lateralis:vastus_latera	GSE10760
8	3	4	52.138	0.00405	heart:sample_was_taken_im	GSE6381
9	3	5	43.448	0.01	vastus_lateralis:inadequate_	GSE8441
10	3	6	37.242	0.0199	skeletal_muscle:24_hours	GSE1295

Supplementary Figure 11: Using formula to define POI in the analysis of MYC and three metabolic pathways (Example IV). The analysis in Figure 7A is similar to using formula-defined POI "$MYC < quantile(MYC, 0.1)$ & $Mitochondria > quantile(Mitochondria, 0.9)$ & $Glycolysis > quantile(Glycolysis, 0.9)$ & $Fattyacid > quantile(Fattyacid, 0.9)$". Here $quantile()$ is the R function for computing quantiles. For example, $quantile(s, 0.9)$ means 90th percentile of the activity of gene set s in all samples. A: Screenshot for the GUI when specifying POI using formula. B: Text box for inputting the formula. C: The enriched biological contexts.

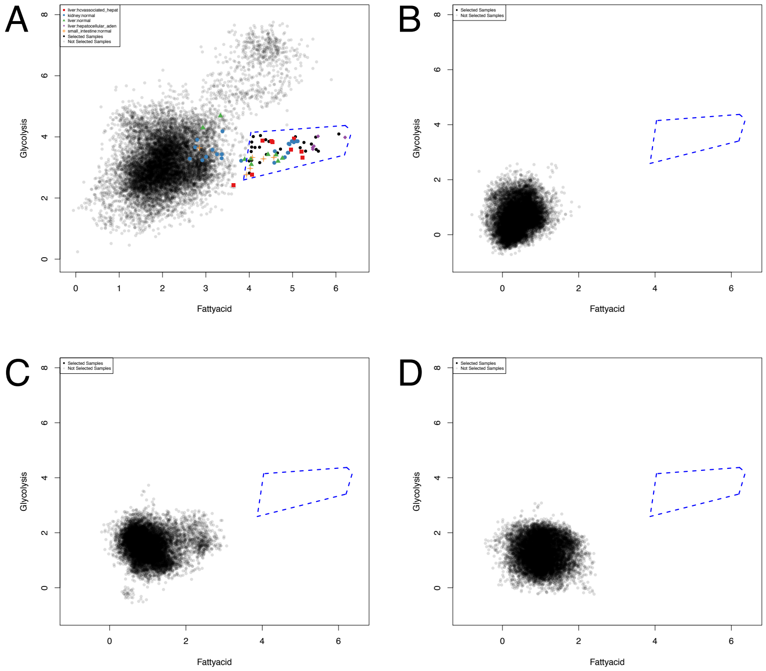

Supplementary Figure 12: Illustration of permuting genes. A: Original gene set activities of glycolysis and fatty acid gene sets. B-D: Gene set activities after permuting genes. Three independent permutations are shown.
